# One Health implementation in Ghana: a perspective on policy development and institutional change

**DOI:** 10.3389/fpubh.2026.1756550

**Published:** 2026-04-02

**Authors:** Ana Maria Perez Arredondo, Katja Bender

**Affiliations:** 1 Center for Development Research (ZEF), University of Bonn, Bonn, Germany; 2 Center for Veterinaty Public Health and One Health, University of Veterinary Medicine Vienna, Vienna, Austria; 3 International Center for Sustainable Development (IZNE) of the University of Applied Science Bonn Rhein-Sieg (HBRS), Siegburg, Germany

**Keywords:** one health implementation, policy analysis, PRISMA, West Africa, narrative analysis

## Abstract

In Ghana, the One Health approach has been recognized as a strategy to strengthen health security. To situate the One Health approach in the prevailing political and institutional structures, this work presents an analysis of the narratives that have accompanied the OH approach in Ghana and enable intersectoral collaborations through the lens of policy change theory. These narratives have implications for steering collective action capacities, strategic foundations, and processes that can drive policy change. Data were collected through reviewing published documents, reports, and gray literature, complemented by interviews and questionnaires with stakeholders. Findings show that existing narratives around One Health policy continue to reinforce institutional silos in planning and implementation. Ghana's One Health policy development represents a significant achievement, having a formalized framework where none existed, and having established mechanisms that bring together previously separate sectors. Yet this study also reveals that current structures may be insufficient for the transformative change that One Health implementation requires. Future research is needed on dimensions that connect policy structures to ground-level realities.

## 1 Introduction

Different approaches have emerged in an attempt to address the complex relationship between health and the environment in a holistic manner. One such approach is One Health (OH), which operates at the intersection of humans, animals and the environment, and encourages integrated, interdisciplinary and multisectoral action (1). Institutionalizing approaches like OH requires different forms of collaboration to be translated into action (2). This complex task begins with achieving coherence across traditionally isolated sectors, and must also consider and include the social, economic and institutional factors that influence health access and availability, as well as largely determining environmental exposures.

Multiple international commitments advocate for the practical implementation of a OH approach and its translation into policy initiatives, such as the International Health Regulations (IHR) (3) and the One Health Joint Plan of Action (OHJPA) (4). These commitments are embedded in long-standing cooperative goals, such as the Alma-Ata

Conference (5), and the Convention on Biological Diversity (6, 7), which aim to mobilize multisectoral expertise. Such commitments have led signatory countries to undergo significant changes at multiple levels, creating concrete actions and pathways for the OH approach to be translated into policies and legislation (8–10). While these frameworks provide important guidance for translating OH into policy and practice, three basic recognitions must be made from a policy analysis perspective. Firstly, the OH approach's holistic nature inherently introduces complexities. Secondly, political reforms are needed for the OH approach to be included in policy initiatives, which may encounter resistance. Thirdly, given the human-animal-environment interdependencies highlighted by the OH approach, the process of institutional change will be highly context-specific.

The article's approach was to build on existing frameworks for institutional and policy change, using narrative analysis for a case study, with the aim of examining the evolution of intersectoral collaboration (ISC) and the institutionalization of OH, in order to investigate the reality of translating OH into policy. Campos and Reich (11–13) have proposed a framework for examining the elements of policy change. However, identifying and interpreting these elements is complex and requires tools for interpretative policy analysis, such as the use of narratives (14–16). These narratives are important for understanding policy change because they form part of broader knowledge production processes within scientific and policy networks (14, 16). Ghana was selected as a case study locale for the *Graduate School of One Health and Urban Transformation*, a collaborative initiative between the University of Bonn and the University of Ghana (17). Moreover, at the time this research commenced, Ghana had recently formed a Technical Working Group (OHTWG), providing a timely opportunity to examine OH governance structures during a critical developmental phase. This combination of existing research infrastructure and emerging policy developments made Ghana an ideal setting for this investigation.

This work has three key contributions. Firstly, it demonstrates how different narratives influence intersectoral collaboration for OH. Secondly, it documents how these narratives influence the stakeholders involved, the capacities for collective action, the strategies for policy change, and policy expectations. And thirdly, the work evaluates the extent to which the current state of ISC could be translated into OH policies.

This study employed a dual analytical approach to examine bottom-up, community-based interventions and top-down, international frameworks that have accompanied the evolution of OH in Ghana (18–20). The bottom-up interventions were identified inductively through a systematic review of academic and non-academic records (21, 22). A few examples from the literature were also identified for the top-down approach. However, the analysis principally focused on the Global Health Security Agenda (GHSA), the overarching framework through which OH implementation advances in the country have been evaluated to support the adherence to the IHR (3, 23). Ghana's capacities that support the IHR have been documented through two successive evaluations, one in 2017 (24) and the second in 2025 (25). The evaluations of the capacities for adhering to the IHR are occurring simultaneously in other signatory countries; therefore, the analysis of the narratives and evolution of OH through the policy change framework makes Ghana a valuable case study and benchmark for other Global South countries in the process of adjusting their institutional landscape to advance the institutionalization of OH, as parallels can be drawn from Ghana's experience.

The initiatives, interventions, capacities, and frameworks accompanying the evolution of OH in Ghana were classified into one of four OH narratives following the categories identified by Galaz et al. (16): holistic integration, risk-surveillance, economic benefits, and extended OH. These narratives were used as analytical tools to understand the motivations for ISC and OH translation into policy. Data were extracted and analyzed using Campos and Reich's policy change framework (11). To validate and refine the findings from the literature review, expert consultations in the form of focus group discussions and one-to-one interviews were conducted.

The section that follows this introduction outlines the analytical framework. The methodology section then provides details of the search strategy and selection criteria used to identify ISC and OH initiatives in Ghana. The results section presents information on the framework elements obtained from the policy change analysis. The discussion section interprets and compares the findings. Finally, the implications for OH policy implementation derived from the review are presented.

## 2 One Health evolution and narratives

### 2.1 Conceptual framework

The conceptual framework reflects the nature of this work, intended to identify the different narratives of OH present in Ghana, to document the stakeholders and actors involved, their specific capacities for collective action, their strategies for policy change, their policy expectations, and to evaluate the extent to which the current OH can be translated into policy.

**Figure 1:**
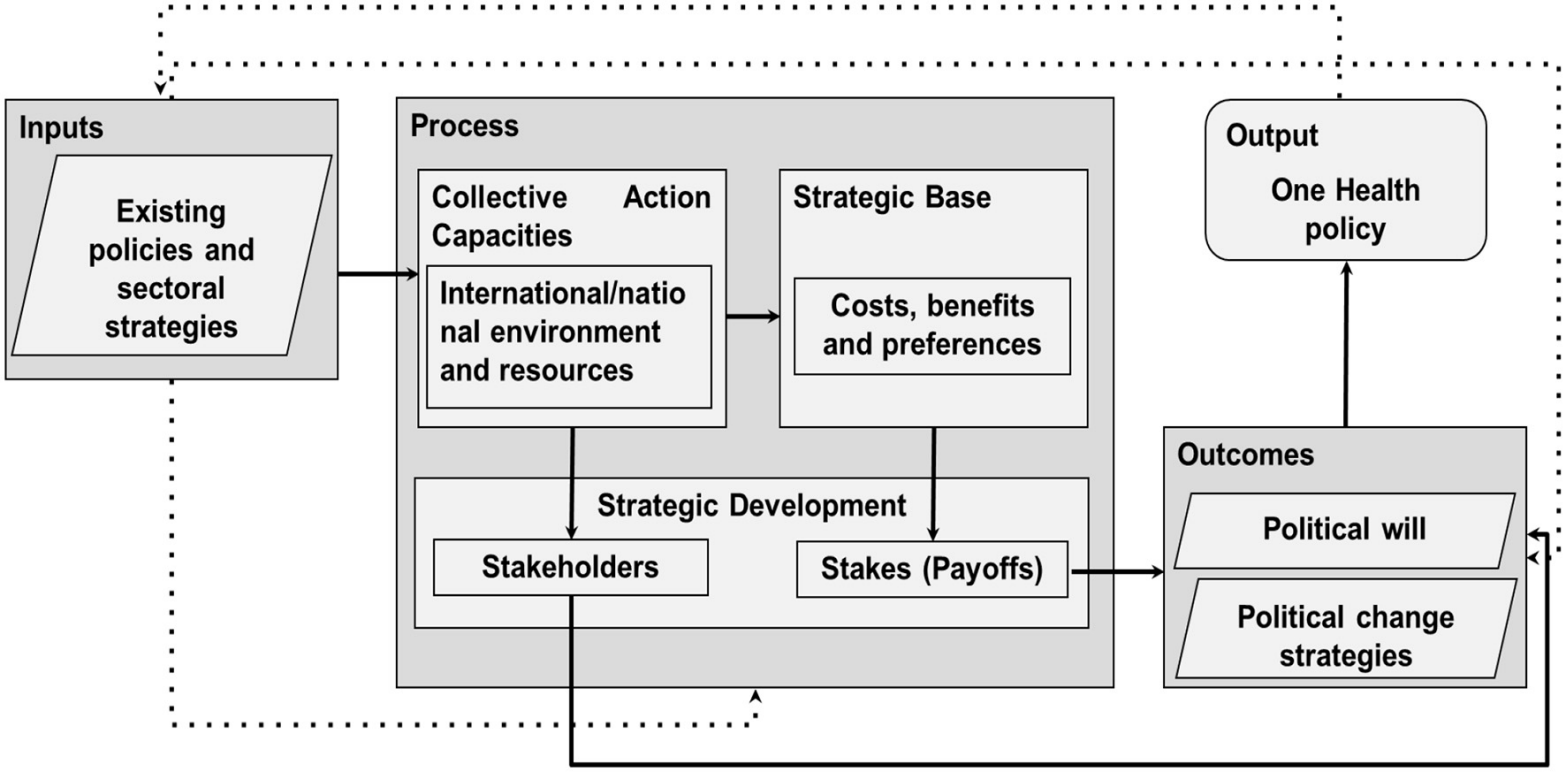
Framework for analysing the structural context of policy developments concerning One Health in Ghana, considering the inputs, process, outcomes and output of policy change.

The framework follows the works of Reich and Campos (11, 12, 26) on the basic elements of reform in health policy, and of Corduneanu-Huci et al. (27) on the structural context in which policy change unfold. These two contributions were combined to create a conceptual framework comprising four blocks. The first of these are the input elements, made up of the existing legislation, policies and strategies at the human-animal-environment interface. The second block, the process, is intended to capture the existing capacities, strategic base and developments for translating OH into policies. The outcome of the process is measured by the existing political will and strategies for continuing the reform. The output is the evaluation of the extent to which current OH perceptions, intentions, and actions can be translated into policies, and informs an iterative process of change (see Figure 1).

In the framework, collective action capacities are the capabilities and constraints that shape collaborative perceptions, intentions, and actions across different levels (27, 28). Specifically for the translation of OH to policies and sectoral strategies, existing policies in public health, food and agriculture, and environmental conservation strongly influence these capacities. The collective action capacities are identified through indicators of 1) the international institutional environment (i.e. constraints from international agreements, donors, and development organizations); 2) the national institutional environment (i.e. constraints shaped by national regulations, acts, action plans, and priorities); and 3) resources (i.e. technical, financial, organizational, and symbolic means that support or oppose institutional change). Operationally, this definition of collective action capacities goes beyond resources and structures, requiring alignment across governance scales of the cognitive (perceptions), motivational (intentions) and behavioral (actions) levels, which links to the strategic base and strategic development.

The strategic base encompasses the immediate incentives or benefits, disincentives or cost, and preferences that shape stakeholder intentions and actions in support (or opposition) to change and are influenced by the collective action capacities. The operational parameters driving stakeholder engagement considered here are societal indicators of socioeconomics, surveillance and prevention, disease emergence, and health environment conditions.

Strategic development, arises from the interaction between collective action capacities and the strategic base, shaping stakeholder perceptions, intentions, and the stakes involved in policy change. It is identified through: 1) stakes or payoffs, reflecting the consequences of the change process for different stakeholders; and 2) stakeholders, comprised by the full range of actors directly or indirectly involved, or affected by, the policy process. The operational parameters reflect complex actor dynamics trough interdependence of health systems, collaboration between health practitioners, integration of sectors, community participation, and societal and demographic change.

The work of Reich and Campos (26) presents the elements above mentioned as policy change context elements, separated from the stakeholder identification and analysis, and the set of political strategies, all of which form part of the Political Analysis. Here however, the outcome elements that emerge from the policy change process serve as prerequisites for policy change, and include: (1) policy strategies, expressed as resources and positions adopted by organizations or individuals engaged in the process; and (2) political will, enacting and sustaining the policy change. Using the political strategies and political will, the last item in the conceptual framework is expressed as an evaluation of the feasibility for the translation of OH to policy in Ghana.

### 2.2 Data and methods for analysis

#### 2.2.1 Research design

To operationalise the conceptual framework, this study employed a multi-tier approach spanning from 2018 to 2025. This temporal sequencing enabled the incorporation of influential national and international perspectives on the development of Ghana's OH policy landscape, the most significant of which was the impact of the global COVID-19 pandemic.

The research design incorporated three distinct yet complementary phases. The first phase, conducted in 2018, involved a scoping review to identify relevant policies and sectoral strategies relating to OH, map collective action capacities, and classify actions and initiatives into OH narratives. The second phase involved contacting stakeholders engaged in ISC relevant to OH, identified from phase 1, for consultation in 2019, with the aim of validating the scoping review findings and gathering information on costs, benefits, preferences, and the stakes or payoffs related to the translation of OH into policy. The third phase involved conducting a systematic literature review in 2025. Each of the phases is described in detail below.

##### 2.2.1.1 Phase 1: initial scoping review (2018)

From January to December 2018, a scoping review of academic and gray literature was conducted following the Preferred Reporting Items for Systematic reviews and Meta-Analyses (PRISMA) guidelines (22, 29, 30). The review aimed to identify and map relevant policies and sectoral strategies relating to ISCs relevant to OH in Ghana and extract information on collaboration narratives and sectoral involvement patterns to map collective action capacities.

Academic literature was identified through Google Scholar and PubMed using the search string [“Ghana” AND (“One Health” OR “Health” OR “Public Health” OR “Agriculture” OR “Environment” OR “Ecosystem” OR “Wildlife” OR “Livestock” OR “Climate”)], on the titles, abstracts and keywords of the documents. The search was limited to English-language documents, including peer-reviewed publications and technical reports. Non-academic literature was retrieved using a snowballing approach from news reports and internet pages of government and international agencies, as well as donor agencies, dealing with public health, animal health and environmental protection.

Inclusion criteria required that documents: (1) demonstrated ISCs, or transdisciplinary focus; (2) addressed the core OH domains (human health, animal health, environmental health); and (3) provided sufficient content to assess ISCs in Ghana. Documents that briefly mentioned Ghana as part of broader analyses were included when they referenced additional sources with substantive Ghana-specific information. Exclusion criteria filtered out documents that lacked sufficient detail to assess ISCs or were not available as open access.

This search identified substantial policy reports, sectoral plans, and policy agendas that informed stakeholder identification and provided the foundation for the two subsequent phases.

##### 2.2.1.2 Phase 2: stakeholder consultation and validation (2019)

Following the identification of narratives and stakeholders from documentary sources, a validation exercise was conducted through stakeholders' consultation. Due to the exploratory nature of the research, a focus group methodology was selected to gather diverse perspectives and validate preliminary findings.

The focus group took place on 21 May 2019 at the Institute of Statistical, Social and Economic Research (ISSER), University of Ghana, organized as part of a workshop by the One Health and Urban Transformation Graduate School. Approximately 50 individuals from government agencies, international organizations, and academic organizations were invited; 29 attended. Participants represented diverse sectors and organizations including ministries, departments, and agencies (MDAs), international organizations, non-governmental organizations (NGOs) and civil society organizations (CSOs), academic organizations, and healthcare facilities.

The 60-min focus group session used open-ended questions to gather information on understanding of OH, resources available for OH collaborations, collective action capacities, expected benefits, aggregation of preferences, and potential implementation challenges. Guiding questions were distributed to participants prior to the event to facilitate reflection (see table 1 in Supplementary material).

To gather deeper insights into OH understandings and motivations for policy change, 18 follow-up semi-structured interviews were conducted from June to December 2019 with representatives of government agencies and international organizations. These interviews provided additional context on policy and sectoral strategies dimensions, institutional barriers, and stakeholder perspectives that informed interpretation of documentary evidence.

##### 2.2.1.3 Phase 3: systematic literature review (2025)

Despite the initial scarcity of records, the exponential growth in academic and gray literature explicitly calling for ISC as a result of the COVID-19 pandemic enabled a systematic review (31–34). The review, in accordance with the PRISMA guidelines (21), was conducted from April to July 2025. The timing of the scoping review and stakeholder consultation, followed by a systematic review, allowed changes on prevalent perspectives on ISC for OH to be monitored. The identification and classification of initiatives, interventions, capacities, strategies, context, and actors, fitted the information categories created inductively in phases 1 and 2 and allowed for a quick assignment into one of the four pre-defined OH narratives from Galaz et al (16).

The literature search was designed to capture literary records relevant to OH evolution in Ghana. PubMed, Scopus, and Web of Science were selected for this exercise. Building on the results from the scoping review, the search string used was:

(Ghana) AND (“One Health” OR “ecohealth” OR “holistic approach^*^” OR “integrated approach” OR “multisectoral” OR “intersectoral” OR “cross-sectoral” OR “transdisciplin^*^” OR “different sectors” OR “multiple sectors” OR “sectoral integration”) AND (“zoonotic diseases” OR zoono^*^ OR rabies OR anthrax OR influenza OR “avian influenza” OR tuberculosis OR schistosomiasis OR “Buruli ulcer” OR “emerging infectious diseases” OR “antimicrobial resistance” OR “pandemic” OR surveillance OR “food safety” OR poultry OR livestock OR “livestock production” OR agricultur^*^ OR aquaculture OR “water borne” OR “water mediated” OR “vector borne” OR ecology^*^ OR “environmental health” OR “ecosystem health” OR “climate change”).

To complement the findings of the academic repository, organizational repositories were consulted to identify policies, reports, sectoral development plans and programme evaluations. These included the Food and Agriculture Organization of the United Nations (FAO) Legal Database (FAOLEX) (35); the World Health Organization (WHO) country repository (36); and the Ghana Legal Information Institute (GhaLII) repository (37). The inclusion and exclusion criteria corresponded to the ones of the scoping review.

After title-abstract screening using Rayyan (38), the full text analysis and data extraction were performed using MAXQDA 24 (39). The table 3 of the Supplementary material provides the detailed clustering between the reported narratives, themes, and the records assigned to each theme.

An updated list of actors was derived from the systematic review results and clustered into five categories: 1) research and academia; 2) international partners (technical and financial); 3) MDAs; 4) NGOs, and CSOs; and 5) other. The remaining elements of the conceptual framework for policy analysis, corresponding to process, outcome, and outputs, were also updated and derived from the extracted data.

#### 2.2.2 Methodological limitations

Several limitations need to be acknowledged. Firstly, the research context was shaped by temporal considerations. In 2018, the OH approach was a niche concept within the Ghanaian political and academic spheres. This limited the evidence that could be collected for the scoping review and meant that the number and scope of the actors involved changed over time. Secondly, while the pandemic brought increased attention to OH in academic work and donor-driven initiatives, it also diverted resources away from broader OH implementation between 2020 and 2023. This could have skewed the observed patterns toward pandemic preparedness and outbreak response. Third, the focus group and interview data reflect the perspectives of the actors who were identified through the initial scoping review and who were eventually engaged with the OH policy processes. Non-mobilized stakeholders, particularly those in sectors indirectly associated with health, remain underrepresented in consultation processes due to difficulties in connecting with them, mainly arising from a lack of awareness or the perceived limited relevance of OH to them. Fourthly, this work relies heavily on document analysis. While these documents represent successful mobilization and official narratives, they may also obscure contested processes and the actual implementation gaps between stated intentions and practice. Finally, reliance on peer-reviewed and open-access documents excluded offline materials and the perspectives of actors less integrated into international knowledge networks. This limitation is particularly relevant given the underrepresentation of certain narratives, which may be due to methodological constraints rather than an actual absence.

## 3 Results

**Figure 2:**
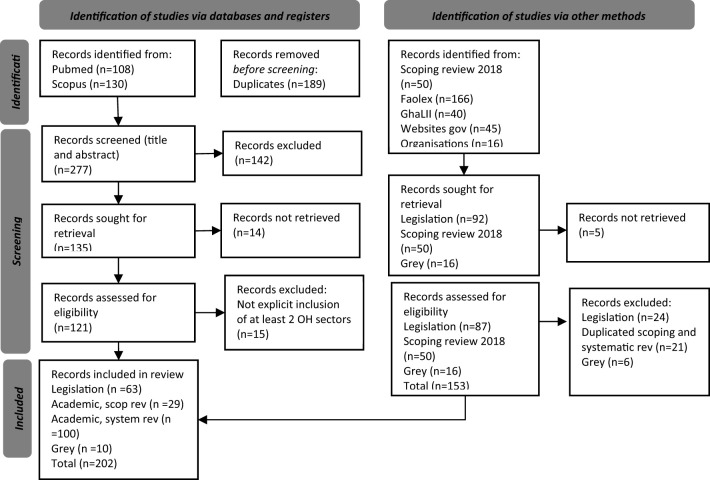
PRISMA flowchart.

The complete selection process is detailed in the PRISMA flowchart (Figure 2).

### 3.1 General temporal and sectoral trends

**Figure 3:**
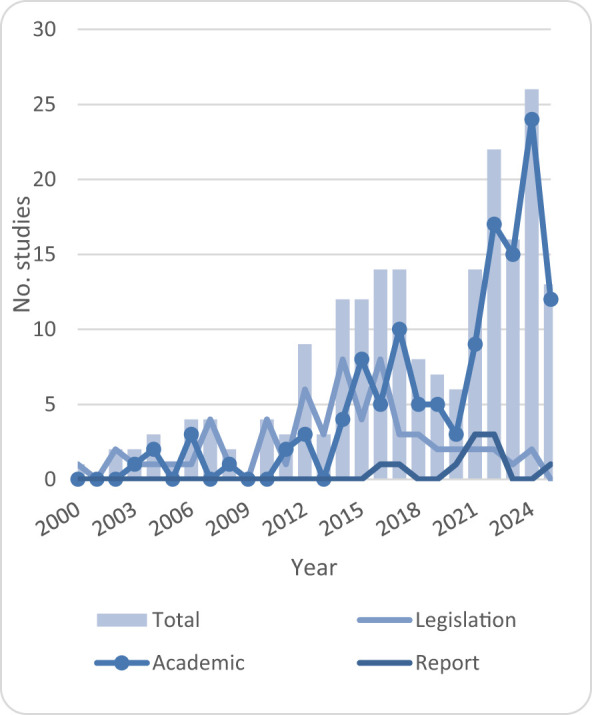
Distribution and trends of publications by year and type.

A total of 202 records were included in the analysis. Of these, 63 were legislation documents, 10 were reports and 129 were academic works. The selected records started in 2000. From 2000 to 2011, fewer than five publications were released per year. There was a positive trend observed in publications reported from 2014 to 2017, which slowed down between 2018 and 2020 before increasing again after 2021. It is important to note that, although the majority of the selected records were academic, legislation recognizing the need for ISC in relation to human, animal and environmental health was on a modest positive trend from 2000 to 2016, stabilizing at a lower level after 2017. This may be a sign that international events kept the production of legislation on hold. It is also relevant to note that expert reports, mostly from international organizations such as the World Bank and various UN agencies, began to emerge from 2016 onwards (Figure 3).

**Figure 4:**
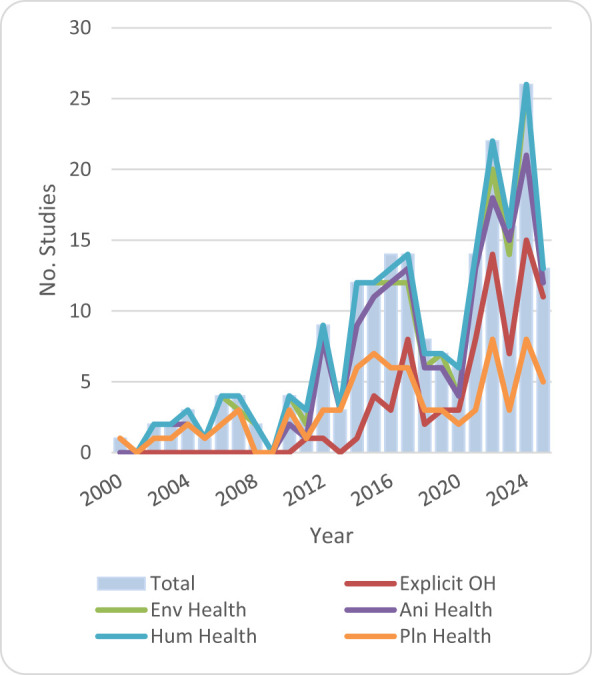
Distribution and trends of publications by year and One Health thematic area.

The sectoral distribution of the reviewed records reveals balanced representation across the three health domains: human health (31%), environmental health (29%), and animal health (27%). An additional category, plant health (13%), was created to accommodate records addressing crop protection, forestry, and plant biodiversity which could not be classified under the other three categories (see Figure 4). The high number of reports on environmental health is due to the fact that environmental health was considered in terms of characteristics that support or hinder the health of humans and animals. It is notable that explicit OH started in 2011 but only became more visible from 2015 onwards (Figure 4).

### 3.2 Elements influencing the policy change process

**Figure 5:**
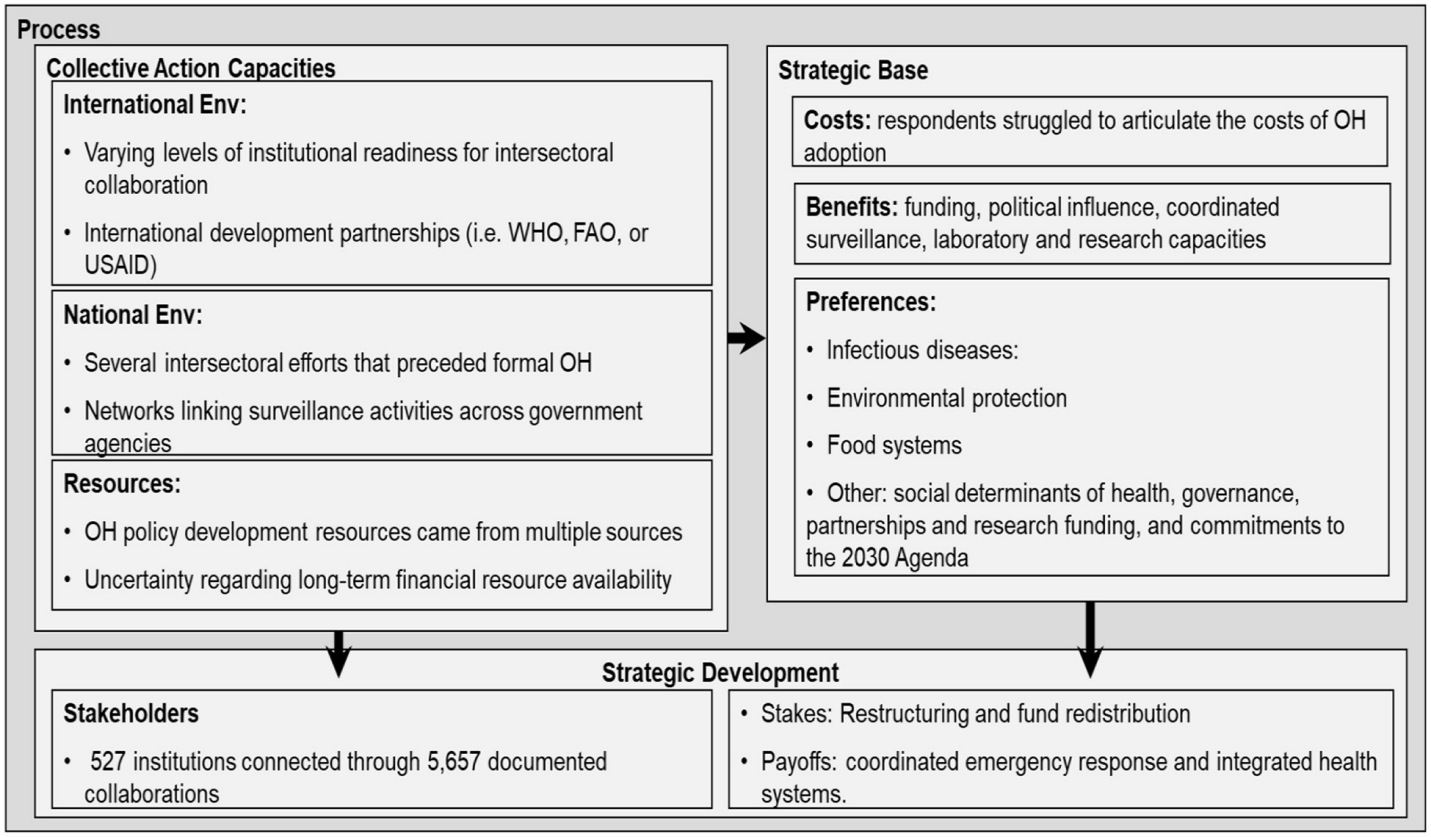
Analysis of the policy change process for One Health in Ghana.

Figure 5 depicts the analytical results concerning the elements forming the policy change process, which are discussed in the subsequent section.

#### 3.2.1 Collective action capacities

##### 3.2.1.1 International environment

International initiatives identified include the 2018 Stepwise Approach toward Rabies Elimination (SARE) assessment by the Global Alliance for Rabies Control (GARC) (40), and the joint effort to conduct Ghana's first dog census by the Breakthrough ACTION project, Rabies in West Africa (RIWA), and AngloGold Ashanti Malaria Control Ltd (AGA Mal) (40–43). Additionally, the COVID-19 pandemic and subsequent outbreaks, such as the 2023 Lassa fever epidemic in Accra, created momentum for new ISC to improve disease surveillance and control (44).

Beyond disease outbreaks, the need for policy change to enable ISC in health was also recognized at the institutional level. Activities that initiated this recognition and established pathways to change included the Global Health Preparedness Program with support from the Norwegian Institute of Public Health (NIPH) (45), the External Evaluation of IHR core capacities (24), and the subsequent zoonoses prioritizing exercise in collaboration with the United States Agency for International Development (USAID) Emerging Pandemic Threats Program (46). The influence of these programmes and collaborations shaped both the discourse around OH in Ghana and the specific priorities emphasized in policy development.

Stakeholders in the consultation exercise reported varying levels of institutional readiness for enhanced ISC. Organizations with prior experience in international development partnerships, particularly those engaging with WHO, FAO, USAID, the Africa Centers for Disease Control and Prevention (Africa CDC), the Global Fund to Fight AIDS, Tuberculosis and Malaria, the United Nations International Children's Emergency Fund (UNICEF), the United States Centers for Disease Control and Prevention (CDC), and the World Bank, expressed greater confidence in OH coordination mechanisms.

##### 3.2.1.2 National institutional environment

Findings from the three phases of data collection demonstrate that, despite ISC and existing avenues connecting two or more institutions working in relevant areas of OH being mentioned in legislation, efforts often remained at the recommendation stage and did not translate into practical implementation. Policy planning, implementation, and evaluation remained predominantly sector-specific, with limited formal channels for information exchange between the sectors of public health, animal health, and environmental protection.

Despite this fragmentation, isolated ISC emerged between complementary agencies. These collaborations typically arose in response to specific challenges rather than as part of a systematic coordination framework. One example was clearly identified with the outbreaks of highly pathogenic avian influenza (HPAI) H5N1 subtype (47–51). The main drivers of ISC were the National Disaster Management Organization (NADMO), operating under the Public Health Act 2012 (No. 851) (52); the Antimicrobial Resistance (AMR) Policy 2017 (53), and the National Food Safety Policy (54). These policy instruments achieved measurable results, including collaboration between National AIDS Control Programme, the National Malaria Control Programme, and the National Tuberculosis Control Programme to improve laboratory system quality and contribute to the Ghana Field Epidemiology and Laboratory Training Programme (GFELTP) (55). Most notably, a shift toward systematic coordination occurred in late 2017 with the establishment of the OHTWG, which in March 2018 conducted a prioritization exercise for zoonotic diseases and developed strategies for disease outbreak prevention and response (46, 51).

Findings from the consultation period corroborated those from the literature review. Stakeholders identified several existing ISCs that pre-dated formal OH discourse in the country. These included disease-specific task forces for rabies and anthrax, the National Action Plan on AMR (50), and informal networks linking surveillance activities across the Ghana Health Service (GHS), the Veterinary Services Directorate (VSD), the Environmental Protection Agency (EPA), research institutions including the University of Ghana and the Kwame Nkrumah University of Science and Technology (KNUST), and civil society partners such as the Ghana Coalition of NGOs in Health. However, organizations primarily focused on sector-specific mandates expressed concerns about capacity constraints and potential mission dilution if the focus were to change to a more extensive OH translation into policies.

##### 3.2.1.3 Resources

The identification of this section of the analytical framework relied on the stakeholder consultation phase. OH policy development resources were provided through multiple channels. Donors, particularly FAO, WHO, USAID, and NIPH, provided technical support and financial resources for OHTWG activities. NADMO provided administrative and organizational resources, hosted regular OHTWG meetings, and established the coordination function within the existing government structure. GHS, VSD, and EPA organized continuous events promoting OH awareness and capacity building. OHTWG members provided crucial political resources, including access to decision-makers, academic organizations, and media for OH advocacy. However, long-term resource availability remained a persistent concern, with heavy reliance on development-cooperation partner funding, limited domestic budget allocation for OH coordination, and human resource contributions dependent primarily on individual initiative rather than institutional commitment.

#### 3.2.2 Strategic base

This section first presents narratives identified from the literature review and stakeholder consultation related to several OH initiatives, interventions, capacities, and frameworks in Ghana to understand the motivations driving ISC and the translation of OH principles into policy. Subsequently, the benefits, costs, and preferences identified during the analysis of data collected from the stakeholder consultation are presented.

Three primary themes were emphasized under Narrative 1 (holistic, integrated approach): the interdependence of health systems and the need to correct siloed approaches to ecosystem and human health (56–59); the necessity for collaboration between health practitioners (55, 60–63); and the integration of disciplines to bridge institutional gaps (18, 64–66). The update from the scoping review to the systematic review revealed that following the COVID-19 pandemic, documents under Narrative 1 showed moderate growth, documenting the formalization of ISC structures and increased institutional engagement with OH frameworks. This growth reflected OHTWG activities and the integration of OH principles into sectoral strategic plans, moving beyond conceptual discussions toward an empirical analysis of coordination mechanisms (51, 67–69).

The records classified under Narrative 2 (strategy to prevent risks and respond to crises) emphasized ISC for surveillance and prevention priorities for emerging diseases in rural areas (56), legal mandates for preventive animal health (57), specific initiatives to reduce Buruli Ulcer infections (60), and national capacities to prevent and respond to public health emergencies (55). Additional focus areas included water and sanitation, particularly concerning irrigation practices and their derived health risks (70), as well as disease outbreaks predominantly zoonoses (61–63, 65, 71–73), neglected tropical diseases (74–80), and AMR (59). The update revealed that over time, records under Narrative 2 reflected an intensified focus on pandemic preparedness, disease surveillance, and emergency response coordination. Specifically, the records on response called for intersectoral coordination mechanisms for case detection and contact tracing, enhanced zoonotic disease and AMR surveillance (69, 81–85), and integration of OH principles into national health security planning (86–88).

Narrative 3 (political and economic benefits) was the least represented in the initial scoping review, with few records reporting on health and socioeconomics (18) or the intersection of science, policy, and political action (58, 89–91). Narrative 3 remained minimally represented in the systematic review, continuing the pattern of limited economic analysis observed in the initial review (92). This persistent gap suggests insufficient attention to cost-effectiveness analyses, economic evaluation of OH interventions, and broader political economy dimensions of OH policy implementation.

The records corresponding to Narrative 4 (social-ecological and local disease context) focused on several key areas. Community participation and development were advanced through analyzing vulnerability and exposure to disease (74, 93), and community-based strategies incorporating local understandings of disease (18, 60, 64, 91, 94). The records also addressed behavioral factors (95), environmental drivers particularly in relation to urbanization, human mobility, and population growth (70, 96), and broader environmental drivers like topography, climatic conditions, and ecosystems, determining livelihood dynamics (97, 98). Despite existing documented research on environmental determinants of health, the updated period yielded only a few additional records emphasizing social-ecological contexts, suggesting continued privileging of biomedical and surveillance-oriented approaches over community-based and ecological frameworks (99–105).

The stakeholder consultation yielded views of OH through three primary lenses: first, as a comprehensive approach to improving health and preventing infectious diseases; second, as an integrated, results-focused framework; and third, as a recognition of the interdependence of animal, human, and environmental health. Most of the stakeholders interviewed situated their institutional work within the human-animal-environment interface, with a focus on human-environment relations. This perception significantly shaped how OH priorities were conceptualized across different institutional mandates, privileging certain health challenges (integration and interdependence at macro level) while potentially marginalizing others (local ecologies, micro level).

Stakeholders consistently identified potential opportunities derived from ISC, including access to international funding options, enhanced political influence, the ability to achieve institutional goals, coordinated surveillance systems, and the development of laboratory and research capacities.

However, a critical finding emerged regarding cost articulation: whilst stakeholders readily identified benefits, they struggled to articulate the costs of OH adoption. This gap suggests that the full implications of policy implementation, particularly regarding resource reallocation and organizational restructuring, had not been thoroughly examined during the development phase. The limited cost articulation may reflect insufficient policy analysis, strategic avoidance of potentially contentious resource allocation discussions, or genuine uncertainty about implementation requirements. Importantly, actors and stakeholders who were non-mobilized or potentially opposed to OH translation into policy opted out of the consultation process, which constrains the critical examination of costs.

Regarding disease and sectoral preferences, results from written reports and stakeholder consultation allowed for identification of several priorities for OH action. Disease focus included tuberculosis, rabies, anthrax, haemorrhagic fevers, HPAI, malaria, and selected neglected tropical diseases (46). Environmental priorities encompassed climate change, protection of natural reserves and wildlife, and Water, Sanitation, and Hygiene (WASH) (24, 25). Food systems priorities included nutrition, food safety, and crop protection. Additional priorities addressed social determinants of health, governance issues including partnerships and research funding, and commitments to the United Nations 2030 Agenda (106, 107). Notably, no clear distinction emerged between urban and rural priorities.

#### 3.2.3 Strategic development

Based on a text analysis of the records included, 527 organizations were identified, connected through 5,657 documented collaborations. Of these, 115 were government agencies, 99 were international partners, and 78 were academic and research organizations, and the rest were classified as other. This pattern reflects the great influence of international OH architecture in shaping implementation, alongside with strong engagement from national agriculture and health sectors.

The identified stakes centered primarily on the reallocation of funding and organizational resources, as restructuring and fund redistribution are required to provide implementation resources and develop capacity for ISCs in OH. The benefits and preferences identified were closely linked to achieving institutional objectives and capitalizing on opportunities from ISC, including simplified and coordinated public health emergency response and the promotion of integrated health systems at the human-animal-environmental interface.

A critical limitation across all phases was that several actors and stakeholders working at the human-animal-environment interface remained immobilized. This fundamentally limited the scope of OH translation into policies and strategies by neglecting important health determinants. Water resources management, meteorological services, land use planning and health financing organizations were identified as non-mobilized stakeholders and subsequently declined to participate in stakeholder consultations when requested to. This represents a particular constraint on addressing the environmental and social determinants of health through OH implementation.

### 3.3 Policy outcomes

**Figure 6:**
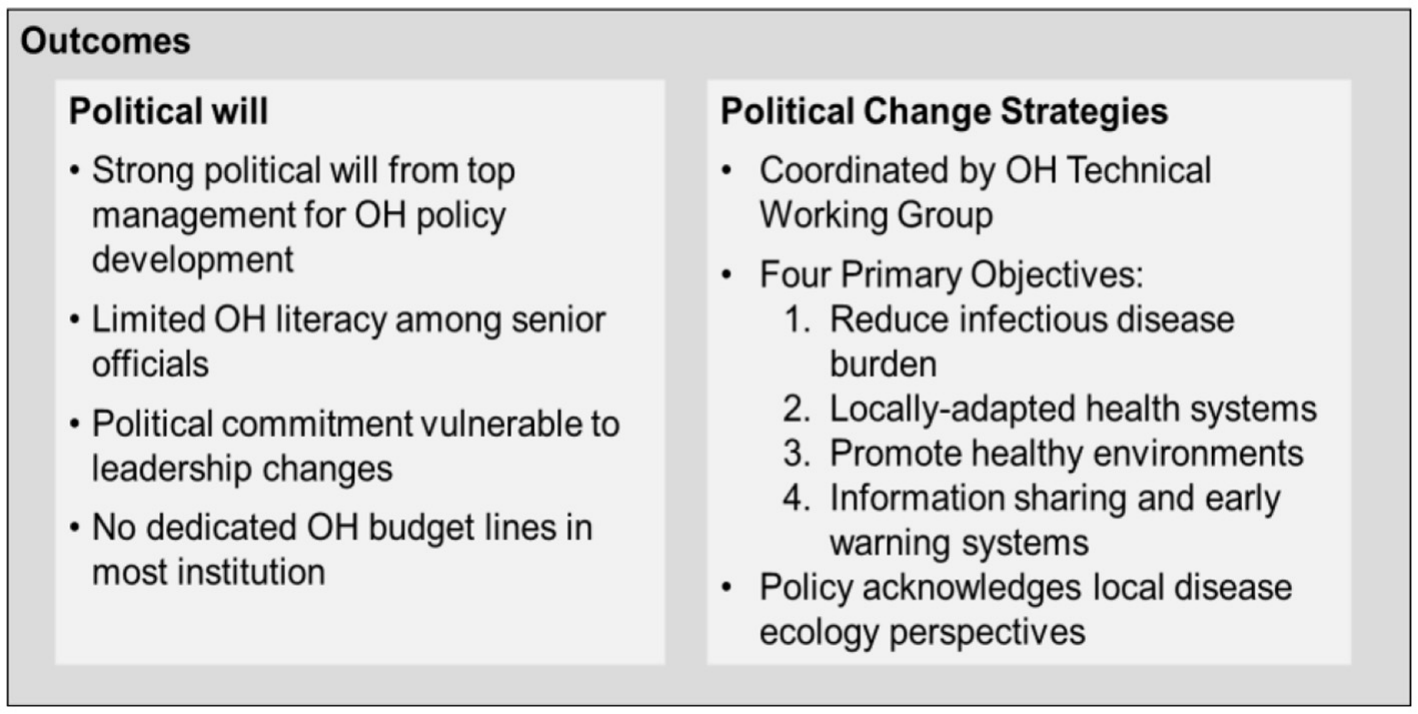
Analysis of the policy change outcomes for One Health in Ghana.

Stakeholder consultations confirmed generally positive political will amongst institutional top management for OH policy development. However, concerns about effective oversight persist due to limited health literacy amongst operational officials and insufficient public incentives for sustained OH engagement. Several stakeholders noted that political commitment remained vulnerable to leadership changes and competing policy priorities, particularly when OH activities required reallocation of resources from established programmes. The absence of dedicated budget lines for OH coordination in most participating organizations reinforces reliance on political goodwill rather than institutionalized commitment (see Figure 6).

The OH policy initiative is coordinated by the OHTWG, an ISC body located within the NADMO with the objective to advancing health security in Ghana through enhanced surveillance and biosecurity enforcement (25). Adoption indicators include development of an integrated health reporting systems and continuous field epidemiology training.

Stakeholder interviews identified four primary policy objectives. First, decreasing morbidity and mortality from infectious diseases by reducing the burden of zoonotic diseases and AMR. Second, achieving local healthy environments and care systems based on local disease ecology understandings. Third, promoting health through incentives for healthy lifestyles and environments, encompassing improved food security, food safety, environmental protection, primary health care provision, veterinary service utilization, and wildlife protection. Fourth, ensuring coordinated, integrated response to disease emergence through efficient information sharing and early warning detection systems.

The articulation of local disease ecology understandings as a policy objective represents notable attention to Narrative 4 perspectives, though as documented earlier, implementation mechanisms for translating this goal into practice remain underdeveloped.

## 4 Discussion

This study examined the evolution, narratives and institutional dynamics that shape OH policy development in Ghana, using a literature review supported by expert consultation. The results highlight the successes of translating OH into policy and initiatives in Ghana, as well as the ongoing challenges to its implementation. While Ghana has successfully established formal OH coordination mechanisms and expanded stakeholder engagement, the findings demonstrate that fundamental tensions remain unresolved and that the influence of international OH narratives and strategies is clear.

The initial scoping phase documented substantial attention to local contexts, as community-based adaptation strategies (93), local understandings of disease-environment relationships (18, 64), environmental determinants of endemic diseases including Buruli ulcer ecology (76, 79, 98), climate factors in cholera transmission (108), and water-borne disease dynamics (74). These works emphasized community perceptions and behaviors, and the intersection of livelihood strategies with disease risk. They represented a body of knowledge grounded in understanding how specific populations in particular ecological contexts experience and respond to health threats. However, with the update to the systematic review and consultation revealed minimal translation of these perspectives into OH policy development. This pattern suggests that the formalization of OH policy, inadvertently narrowed focus toward standardized, internationally aligned surveillance and reporting mechanisms. The influence of the IHR and the GHSA shaped what counts as OH-relevant activity.

The dominance of crisis-response framing across all phases of the reported records represents both the strength and limitations of OH development in Ghana. This pattern is not coincidental, but reflects political, sectoral reforms and institutional changes driven by emergent threats. However, crisis framing has inherent limitations, which became evident during the stakeholder consultation phase. When organizations primarily perceive OH as emergency response infrastructure, three problematic patterns emerge. Firstly, engagement becomes episodic rather than sustained, with collaboration intensifying during crises. Secondly, investment focuses on surveillance and reporting systems, neglecting the factors that cause disease emergence. Thirdly, the urgency of crisis response provides an excuse to avoid difficult conversations about the need for institutional restructuring. The stakeholder consultations illuminated this dynamic directly. While participants readily articulated the benefits of OH in times of crisis, they struggled to identify its costs beyond vague references to limited resources and coordination challenges. The literature review revealed that the focus has shifted beyond pure crisis response, with a growing recognition that OH is an institutional reform requiring sustained political commitment rather than merely emergency coordination. The development of AMR surveillance platforms, the adoption of OH principles in the National Food Safety Policy and the emergence of collaborative research networks addressing human-animal-environment linkages represent the first steps toward this evolution direction.

From the perspective of the stakeholders interviewed, the benefits of OH were articulated with striking consistency and enthusiasm. These included opportunities for multi-stakeholder partnerships, increased access to funding, enhanced political influence, coordinated surveillance capacity, development of laboratory and research infrastructure, and fulfillment of international health security commitments. This convergence around the anticipated benefits helps to explain the political feasibility of OH policy development, as supporters could unite around these shared opportunities. However, in sharp contrast, the costs remained poorly defined. References to limited financial and human resources, different understandings of OH, unspecified laws and regulations, and generic coordination problems lack the specificity that would enable serious negotiation and problem solving. This asymmetry between the benefits that have been articulated and the costs that have not reveals that the redistribution of responsibilities, authorities and resources across sectors has been systematically avoided rather than addressed. This prompts speculation that the most consequential finding from the stakeholder consultation phase may be what was not discussed: the costs, trade-offs and structural implications of OH adoption.

### 4.1 Policy implications: from development to implementation

The transition of OH to policy and strategies presents a critical juncture. Five strategic priorities emerge from the findings, each targeting specific gaps revealed through the analysis.

#### 4.1.1 Confronting policy change

Moving beyond voluntary coordination requires explicit negotiation of costs, authorities, and accountabilities. This demands structured dialogue among OHTWG members and relevant non-participants to identify 1) which institutional functions logically belong at sectoral vs. intersectoral levels; 2) how budgets can be restructured to support genuine joint activities rather than parallel sectoral funding; what accountability mechanisms will govern ISC without creating bureaucratic paralysis; and 3) which legislative reforms would enable rather than constrain integration.

#### 4.1.2 Rebalancing crisis response and prevention

Without diminishing the importance of surveillance and outbreak response, OH policy must expand to address social and environmental determinants of health. This requires bringing currently non-mobilized stakeholders into meaningful roles rather than token participation. Specific strategies should connect OH objectives to the core mandates of peripheral organizations.

#### 4.1.3 Institutionalizing local ecological perspectives

Institutionalizing local ecological perspectives. In order to address the marginalization of local ecologies and community-based approaches, there must be deliberate mechanisms in place to incorporate local knowledge into OH policy processes. This involves establishing formal channels through which community health workers, traditional medicine practitioners and civil society organizations can inform surveillance priorities and intervention designs, developing participatory risk assessment methodologies to complement technical epidemiological analysis, and creating funding streams for community-based research into disease ecologies and the relationships between health and the environment. There is a long-standing tradition of this work, with multiple examples; the challenge is to find ways of incorporating it into current national priorities.

#### 4.1.4 Developing sustainable financing architecture

Heavy reliance on development partner funding creates vulnerability and limits domestic ownership. Sustainable OH implementation requires identifying domestic revenue sources, whether through explicit OH budget lines in participating ministries, pooled funding mechanisms drawing from multiple sectoral budgets, health insurance system contributions, or innovative financing leveraging private sector partnerships aligned with OH objectives.

#### 4.1.5 Establishing implementation monitoring and adaptive learning

The analytical approach documented here provides a baseline for tracking the evolution of OHTWGs. Future monitoring should examine whether crisis-response framing remains dominant, or whether systemic approaches gain traction. It should also examine how non-mobilized stakeholders, identified in this analysis, become engaged, or remain peripheral. Monitoring should also examine whether voluntary OHTWG coordination evolves toward mandated structures, whether the gap between broad policy goals and narrow surveillance indicators persists, and how power dynamics, resource flows and accountability mechanisms develop during implementation.

Beyond these specific strategies, broader questions merit attention in future research and policy dialogue:

- How do different models of ISC perform under various conditions (i.e. global strategies and local realities)?
- How can traditional organizations and native knowledge systems be included?
- What governance innovations might reconcile the need for rapid emergency response with sustained attention to the different determinants of health?

### 4.2 Methodological considerations and study limitations

The analytical approach provided complementary perspectives that strengthened this investigation. The initial scoping phase documented that Ghana had substantive experience with ISC even when not explicitly labeled as OH. The stakeholder consultation phase captured perspectives during active policy development, revealing gaps in cost articulation and tensions between rhetoric and structural change that might not have been evident from document analysis alone. The systematic review enabled the assessment of how OH translation into policy evolved and changed over time. However, several limitations need to be recognized. The stakeholder consultation phase employed convenience sampling through a policy development workshop, likely overrepresenting already-engaged actors while underrepresenting peripheral or skeptical voices. The voluntary participation in interviews may have biased toward more enthusiastic respondents, potentially explaining the positive political will reports despite structural evidence of limited change. The post-COVID-19 period remains brief, limiting assessment of whether observed patterns represent sustained shifts. Additionally, reliance on published documents and reported perspectives rather than direct observation of coordination practices means the analysis captures formal structures and stated intentions more readily than actual implementation processes. Finally, this study examined policy development more thoroughly than implementation. The findings on structural tensions, cost avoidance, and marginalized perspectives predict challenges that will emerge during implementation, but actual observation of how these tensions play out in practice awaits future research.

## 5 Conclusion

This analysis, based on comprehensive documentary review and stakeholder consultations, reveals a complex landscape where institutional dynamics, evolving narratives, and emerging collaborative structures intersect. Through the lens of policy change theory, this study has traced the trajectory of OH from scattered intersectoral initiatives to a formalized policy framework, exposing both the achievements and paradoxes inherent in this evolution.

Ghana's OH evolution represents a significant achievement, having a formalized framework where none existed, and having established mechanisms that bringing together previously separate sectors. The OHTWG and associated structures embody genuine commitment from diverse stakeholders and reflect substantial effort to translate global OH principles into national context.

Yet this study also reveals that current structures may be insufficient for the transformative change that OH implementation requires. Ghana's experience suggests that successful OH implementation will require not only technical coordination and capacity building but also political will to confront the costs of reform, new institutional constellations, and an epistemological change to make room for approaches that do not fit neatly into the existing frameworks.

Future research is needed on dimensions that connect policy structures to ground-level realities, as the determinants of health behavior in contexts where OH interventions are implemented, the economic implications of environmental conservation for communities whose livelihoods depend on natural resource use, and the effectiveness of different models for community engagement in OH approaches. Understanding these connections would strengthen the evidence base for implementation while potentially revealing why locally grounded approaches have been marginalized and what would be required to restore their prominence.
