# Supplementary material for: One Health implementation in Ghana: a perspective on policy development and institutional change
Source: Front Public Health. 2026 Apr 2;14:1756550. doi: 10.3389/fpubh.2026.1756550 (PMC13083099; doi:10.3389/fpubh.2026.1756550)
Supplement: Supplementary file 1 [file Table_1.docx]

# Supplementary Materials

Table 1 Guiding questions for focus group discussion and follow-up interviews

| OH understanding | - Are you familiar with the OH concept? How would you describe it? |
| --- | --- |
| Stakes (Payoffs) | - In which ways is (or could) your Institution benefiting from adopting an approach that looks at the Animal-Human-Environment interface, or at least on two of these areas? |
| Resources | - Do you think there is enough will from the directors of your institution to further develop (or explore) the OH approach? |
| Preference intensity | - Would you consider that working at the Animal-Human-Environment interface (or at least two of these three areas) is an exogenous concept to your Institute, or do you think that the nature of the work your organisation caries has always been covering it? |
| Aggregation of preferences | - Which is (or could be) the comparative advantage that your Institution offers to current or potential partners interested in adopting an approach that looks at the Animal-Human-Environment interface, or at least on two of these areas? - Imagine that your institute succeeds to implement an approach that looks at the Animal-Human-Environment interface, or at least on two of these areas. Which objectives would it be serving? |
| Collective action capacities | - Which are (or could be) the most relevant working partners for your Institution in order to succeed in taking an approach that looks at the Animal-Human-Environment interface, or at least on two of these areas? - Which are (or could be) the biggest challenges for your Institution in order to partner with other organizations for taking an approach that looks at the Animal-Human-Environment interface, or at least on two of these areas? - What is (or could) facilitating the collaborations for taking an approach that looks at the Animal-Human-Environment interface, or at least on two of these areas? |

| Table 2. List of records included in the systematic review | |
| --- | --- |
| Nr | Citation/Title of Policy |
| 1 | Abbiw, R. K., Mensah, G. I., Adabie-Gomez, D. A. M., Asare-Dompreh, K., Clement-Owusu, S., Adjei, V. Y., Simpson, S. V., Ahmed, M. A., & Johnson, S. A. M. (2024). Seroprevalence of Q fever (Coxiella burnetii) in sheep in the Kwahu West municipality, Eastern Region, Ghana. Heliyon, 10(12). https://doi.org/10.1016/j.heliyon.2024.e33009 |
| 2 | Abd El Wahed, A., Kadetz, P. I., Okuni, J. B., Dieye, Y., Frimpong, M., Ademowo, G. O., Makiala-Mandanda, S., Woldeamanuel, Y., Eltom, K. H., & Yeboah, G. B. (2024). An African One Health network for antimicrobial resistance and neglected tropical diseases. Nature Medicine, 30(1), 10–11. https://doi.org/10.1038/s41591-023-02666-0 |
| 3 | Abdulai, P. M., Sam, K., Onyena, A. P., Ezejiofor, A. N., Frazzoli, C., Ekhator, O. C., Udom, G. J., Frimpong, C. K., Nriagu, J. O., & Orisakwe, O. E. (2024). Persistent organic pollutants and heavy metals in Ghanaian environment: A systematic review of food safety implications. Environmental Monitoring and Assessment, 196(4). https://doi.org/10.1007/s10661-024-12500-w |
| 4 | Acheampong, E. N. (2022). Toward an Integrated Approach: Water Management Dynamics in the City of Accra. Sustainable Development Goals Series, 69–83. https://doi.org/10.1007/978-3-030-95979-1_5 |
| 5 | Ackumey, M. M., Kwakye-Maclean, C., Ampadu, E. O., de Savigny, D., & Weiss, M. G. (2011). Health services for Buruli ulcer control: lessons from a field study in Ghana. PLoS neglected tropical diseases, 5(6), e1187. |
| 6 | Adeapena, W., Afari-Asiedu, S., Najjemba, R., van Griensven, J., Delamou, A., Buabeng, K. O., & Asante, K. P. (2021). Antibiotic Use in a Municipal Veterinary Clinic in Ghana. Tropical Medicine and Infectious Disease, 6(3). https://doi.org/10.3390/tropicalmed6030138 |
| 7 | Adebowale, O. O. (2024). Bacterial zoonoses: Anthrax. 128–138. https://doi.org/10.1079/9781800622852.0009 |
| 8 | Adomako, Boakye-Yiadom; Baiden, Frank; Sackey, Samuel; Ameme, Donne Kofi; Wurapa, Fred; Nyarko, Kofi Mensah et al. (2018): Dog Bites and Rabies in the Eastern Region of Ghana in 2013-2015: A Call for a One-Health Approach. In Journal of tropical medicine 2018, p. 6139013. DOI: 10.1155/2018/6139013. |
| 9 | Adomako, L. A. B., Yirenya-Tawiah, D., Nukpezah, D., Abrahamya, A., Labi, A.-K., Grigoryan, R., Ahmed, H., Owusu-Danquah, J., Annang, T. Y., Banu, R. A., Osei-Atweneboana, M. Y., Timire, C., Tweya, H., Ackon, S. E. D., Nartey, E., & Zachariah, R. (2021). Reduced Bacterial Counts from a Sewage Treatment Plant but Increased Counts and Antibiotic Resistance in the Recipient Stream in Accra, Ghana—A Cross-Sectional Study. Tropical Medicine and Infectious Disease, 6(2), 79. https://doi.org/10.3390/tropicalmed6020079 |
| 10 | Adzitey, F., Huda, N., & Mohd Shariff, A. H. (2021). Phenotypic antimicrobial susceptibility of escherichia coli from raw meats, ready-to-eat meats, and their related samples in one health context. Microorganisms, 9(2), 1–11. https://doi.org/10.3390/microorganisms9020326 |
| 11 | Agyarkwa, M. A., Azaglo, G. S. K., Kokofu, H. K., Appah-Sampong, E. K., Nerquaye-Tetteh, E. N., Appoh, E., Kudjawu, J., Worlanyo, E., Batong, M. F., Akumwena, A., Labi, A. K., Osei, M. M., Satyanarayana, S., Terry, R. F., Manzi, M., & Opintan, J. A. (2022). Surveillance of WHO Priority Gram-Negative Pathogenic Bacteria in Effluents from Two Seafood Processing Facilities in Tema, Ghana, 2021 and 2022: A Descriptive Study. International journal of environmental research and public health, 19(17), 10823. https://doi.org/10.3390/ijerph191710823 |
| 12 | Ahmed, H., Zolfo, M., Williams, A., Ashubwe-Jalemba, J., Tweya, H., Adeapena, W., Labi, A. K., Adomako, L. A. B., Addico, G. N. D., Banu, R. A., Akrong, M. O., Quarcoo, G., Borbor, S., & Osei-Atweneboana, M. Y. (2022). Antibiotic-Resistant Bacteria in Drinking Water from the Greater Accra Region, Ghana: A Cross-Sectional Study, December 2021-March 2022. International journal of environmental research and public health, 19(19), 12300. https://doi.org/10.3390/ijerph191912300 |
| 13 | Akenten, C. W., Ofori, L. A., Khan, N. A., Mbwana, J., Sarpong, N., May, J., Thye, T., Obiri-Danso, K., Paintsil, E. K., Fosu, D., Philipps, R. O., Eibach, D., Krumkamp, R., & Dekker, D. (2023). Prevalence, Characterization, and Antimicrobial Resistance of Extended-Spectrum Beta-Lactamase-Producing Escherichia coli from Domestic Free-Range Poultry in Agogo, Ghana. FOODBORNE PATHOGENS AND DISEASE, 20(2), 59–66. https://doi.org/10.1089/fpd.2022.0060 |
| 14 | Akorli, E.A., Andoh, N.E., Egyirifa, R.K. et al. (2024). Mosquito breeding water parameters are important determinants for Microsporidia MB in the aquatic stages of Anopheles species. Parasites & Vectors, 17(1), 509. https://doi.org/10.1186/s13071-024-06596-9 |
| 15 | Alves, A. C. N., de Almeida Santos, A. N., Dos Santos, S. M., Carvalho, J. R., Rufino, J. P. F., & de Oliveira, A. T. (2025). Mercury Level in Worldwide Poultry Food Products: A Systematic Review. Biological Trace Element Research. https://doi.org/10.1007/s12011-025-04659-1 |
| 16 | Amegah, Adeladza K.; Jaakkola, Jouni Jk; Quansah, Reginald; Norgbe, Gameli K.; Dzodzomenyo, Mawuli (2012): Cooking fuel choices and garbage burning practices as determinants of birth weight: a cross-sectional study in Accra, Ghana. In Environmental health : a global access science source 11, p. 78. DOI: 10.1186/1476-069X-11-78. |
| 17 | Amissah-Reynolds, P. K., Yamoah, J. A. A., Abonie, S. D., Effah-Yeboah, E., Ofori, S. A., Agyei, V., Danquah, J. B., & Kongkuah, C. (2023). Zoonotic Parasites From Dogs In Different Agroecological Zones In Ghana. Journal of the Ghana Science Association, 21(2), 81–90. |
| 18 | Amoah, L. A. O., Oppong, M., Amoah, S. K., & Bimi, L. (2023). Toxocariasis in Ghanaian neighbourhoods: A need for action. Science in One Health, 2. https://doi.org/10.1016/j.soh.2023.100018 |
| 19 | Amoah, P., Drechsel, P., & Abaidoo, R. C. (2005). Irrigated urban vegetable production in Ghana: Sources of pathogen contamination and health risk elimination. Irrigation and Drainage, 54, S49–S61. https://doi.org/10.1002/ird.185 |
| 20 | Amuasi, G. R., Dsani, E., Owusu-Nyantakyi, C., Amoa-Owusu, F., Mohktar, Q., Nilsson, P., Adu, B., Hendriksen, R. S., & Egyir, B. (2023). Enterococcus species: Insights into antimicrobial resistance and whole-genome features of isolates recovered from livestock and raw meat in Ghana. Frontiers in Microbiology, 14. https://doi.org/10.3389/fmicb.2023.1254896 |
| 21 | Anokye, K., Mohammed, A. S., Agyemang, P., Agya, B. A., Amuah, E. E. Y., & Sodoke, S. (2024). A systematic review of the impacts of open burning and open dumping of waste in Ghana: A way forward for sustainable waste management. Cleaner Waste Systems, 8. https://doi.org/10.1016/j.clwas.2024.100152 |
| 22 | Aryeetey, R. N. O., & Coomson, J. B. (2022). Rapid review of key policies and programs linked with nutrition and health in Ghana. African Journal of Food, Agriculture, Nutrition and Development, 22(2), 19727–19777. https://doi.org/10.18697/ajfand.107.21825 |
| 23 | Aryeetey, R. N. O., & Ramos, A. I. (2022). Process and lessons learned in the development of food-based dietary guidelines in Ghana. African Journal of Food, Agriculture, Nutrition and Development, 22(2), 19702–19726. https://doi.org/10.18697/ajfand.107.21830 |
| 24 | Asiedu-Berkoe, F., Chandi, G. M., Bandoh, D. A., Atsu, B. K., Lokossou, V. K., Antara, S. N., ... & Kenu, E. (2022). State of public health emergency preparedness and response capacity of Ghana. Journal of Interventional Epidemiology and Public Health, 5(4), 1-16. |
| 25 | Asuming-Bediako, N., Kunadu, A. P. H., Jordan, D., Abraham, S., & Habib, I. (2022). Prevalence and antimicrobial susceptibility pattern of Campylobacter jejuni in raw retail chicken meat in Metropolitan Accra, Ghana. International Journal of Food Microbiology, 376. https://doi.org/10.1016/j.ijfoodmicro.2022.109760 |
| 26 | Asuming-Bediako, N., Kunadu, A. P.-H., Abraham, S., & Habib, I. (2019). Campylobacter at the Human-Food Interface: The African Perspective. PATHOGENS, 8(2). https://doi.org/10.3390/pathogens8020087 |
| 27 | Awoonor-Williams, John; Apanga, Paschal; Anyawie, Maurice; Abachie, Thomas; Boidoitsiah, Stephen; Opare, Joseph; Adokiya, Martin (2016): Anthrax Outbreak Investigation among Humans and Animals in Northern Ghana: Case Report. In IJTDH 12 (2), pp. 1–11. DOI: 10.9734/IJTDH/2016/22359. |
| 28 | Baah, D. A., Kotey, F. C. N., Dayie, N. T. K. D., Codjoe, F. S., Tetteh-Quarcoo, P. B., & Sampane-Donkor, E. S. (2022). Multidrug-Resistant Gram-Negative Bacteria Contaminating Raw Meat Sold in Accra, Ghana. Pathogens, 11(12). https://doi.org/10.3390/pathogens11121517 |
| 29 | Bandoh, D. A. B., Kenu, E., Dwomoh, D., Afari, E. A., & Dzodzomenyo, M. (2024). A study to evaluate WASH interventions and risk factors of diarrhoea among children under five years, Anloga district, Ghana: A research protocol. PLOS ONE, 19(5). https://doi.org/10.1371/journal.pone.0302754 |
| 30 | Bardosh, Kevin Louis; Ryan, Sadie J.; Ebi, Kris; Welburn, Susan; Singer, Burton (2017a): Addressing vulnerability, building resilience: community-based adaptation to vector-borne diseases in the context of global change. In Infectious diseases of poverty 6 (1), p. 166. DOI: 10.1186/s40249-017-0375-2. |
| 31 | Bardosh, Kevin Louis; Scoones, Jake Cornwall; Grace, Delia; Kalema-Zikusoka, Gladys; Jones, Kate E.; Balogh, Katinka de et al. (2017b): Engaging research with policy and action: what are the challenges of responding to zoonotic disease in Africa? In Philosophical transactions of the Royal Society of London. Series B, Biological sciences 372 (1725). DOI: 10.1098/rstb.2016.0172. |
| 32 | Basu, N. (nil), Clarke, E. E. K., Green, A., Calys-Tagoe, B. N. L., Chan, L. H. M., Dzodzomenyo, M., Fobil, J. N., Long, R. N., Neitzel, R. L., & Obiri, S. A. (2015). Integrated Assessment of Artisanal and Small-Scale Gold Mining in Ghana-Part 1: Human Health Review. International Journal of Environmental Research and Public Health, 12(5), 5143–5176. https://doi.org/10.3390/ijerph120505143 |
| 33 | Basu, N. (nil), Renne, E. P., & Long, R. N. (2015). An integrated assessment approach to address artisanal and small-scale gold mining in Ghana. International Journal of Environmental Research and Public Health, 12(9), 11683–11698. https://doi.org/10.3390/ijerph120911683 |
| 34 | Bell J.A., Nuzzo J.B. (2021) Global Health Security Index: Advancing Collective Action and Accountability Amid Global Crisis, Ghana. Country Score Justification, 2021. Available: www.GHSIndex.org |
| 35 | Blaizot, R., Pasquier, G., Koné, A. K., Duvignaud, A., & Demar, M. P. (2024). Cutaneous leishmaniasis in sub-Saharan Africa: A systematic review of Leishmania species, vectors and reservoirs. Parasites and Vectors, 17(1). https://doi.org/10.1186/s13071-024-06381-8 |
| 36 | Boafo, Y. A., Ohemeng, F. N. A., Ayivor, J., Ayitiah, J. A., Yirenya-Tawiah, D., Mensah, A., Datsa, C., Annang, T. Y., & Adom, L. (2024). Unraveling diarrheal disease knowledge, understanding, and management practices among climate change vulnerable coastal communities in Ghana. Frontiers in public health, 12, 1352275. https://doi.org/10.3389/fpubh.2024.1352275 |
| 37 | Boon, E. K. (2019). Urban environmental health management challenges and prospects in Ghana: A case study of the Accra metropolis. Journal of Human Ecology, 65(1), 26–40. https://doi.org/10.31901/24566608.2019/65.1-3.3136 |
| 38 | Calland, J. K., Haukka, K., Kpordze, S. W., Brusah, A., Corbella, M., Merla, C., Samuelsen, O., Feil, E. J., Sassera, D., Karikari, A. B., Saba, C. K. S., Thorpe, H. A., & Corander, J. (2023). Population structure and antimicrobial resistance among Klebsiella isolates sampled from human, animal, and environmental sources in Ghana: A cross-sectional genomic One Health study. Lancet Microbe, 4(11), e943–e952. https://doi.org/10.1016/S2666-5247(23)00208-2 |
| 39 | Chukwuka, A. V., Adegboyegun, A. D., & Adeogun, A. O. (2025). Algal bloom-mediated microplastic dispersion in coastal areas of West Africa: Integrated insights and risk projections from molecular models and remote-sensed evaluations. Journal of Hazardous Materials, 489. https://doi.org/10.1016/j.jhazmat.2025.137590 |
| 40 | Clarke, Edith Essie(2004). The experience of starting a poison control centre in Africa–the Ghana experience. Toxicology, 198(1), 267–272. https://doi.org/10.1016/j.tox.2004.02.001 |
| 41 | Codjoe, Samuel Nii Ardey; Larbi, Reuben Tete (2016): Climate change/variability and schistosomiasis transmission in Ga district, Ghana. In Climate and Development 8 (1), pp. 58–71. DOI: 10.1080/17565529.2014.998603. |
| 42 | Colecraft, E. K., Marquis, G. S., & Pinto, C. M. (2022). Growing and Learning Together in Fostering Multisectoral Participation for Sustaining Interventions: Lessons from 3 Successive Integrated Multidisciplinary Interventions in Rural Ghana. Current Developments in Nutrition, 6(9). https://doi.org/10.1093/cdn/nzac124 |
| 43 | Co-management Policy for the Fisheries Sector |
| 44 | Constantin de Magny, G., Cazelles, B., & Guégan, J. F. (2006). Cholera threat to humans in Ghana is influenced by both global and regional climatic variability. EcoHealth, 3(4), 223–231. https://doi.org/10.1007/s10393-006-0061-5 |
| 45 | Dakubo, Crescentia (2004): Ecosystem Approach to Community Health Planning in Ghana. In EcoHealth 1 (1), pp. 50–59. DOI: 10.1007/s10393-004-0001-1. |
| 46 | Dakubo, Crescentia (2006): Applying an ecosystem approach to community health research in rural Northern Ghana. |
| 47 | Damba, O. T., Ageyo, C. O., Kizito, F., Mponela, P., Yeboah, S., Clottey, V. A., Oppong-Mensah, B. A., Bayala, J., Adomaa, F. O., Dalaa, M. A., Martey, F., Huyer, S., Zougmore, R., Tepa-Yotto, G., & Tamo, M. (2024). Constructing A Climate-Smart readiness index for smallholder farmers: The case of prioritized bundles of climate information services and climate smart agriculture in Ghana. CLIMATE SERVICES, 34. https://doi.org/10.1016/j.cliser.2024.100453 |
| 48 | Dandi, S. O., Evensen, Ø., Addo, S., Abarike, E. D., Abobi, S. M., Doke, D. A., Lyche, J. L., Mutoloki, S., Obiakara-Amaechi, A. I., Cudjoe, K. S., & Edziyie, R. E. (2025). Antibiotics governance in aquaculture: knowledge, practices, and challenges among stakeholders on the Volta Lake in Ghana. One health outlook, 7(1), 22. https://doi.org/10.1186/s42522-025-00141-z |
| 49 | Darteh, B. A., Cofie, O. O., Nikiema, J., Mapedza, E., Gebrezgabher, S. A., & Okem, A. E. (2023). Response to COVID-19: Building resilience through water and wastewater management in Ghana. Journal of Water Sanitation and Hygiene for Development, 13(10), 811–824. https://doi.org/10.2166/washdev.2023.112 |
| 50 | Dogbey, G., Unim, B., Kwamena, A. R., Ahiabor, G. C., Kisiel, M. A., Zhou, X., Maima, A. O., Abdul-Aziz, A., Enyetornye, B., Niayele, R., Agbolosu, A. A., & Asare-Dompreh, K. (2025). Assessing the Knowledge and Experience of Healthcare Workers in Zoonoses and the One Health Approach: A Cross-Sectional Study in Ghana. ENVIRONMENTAL HEALTH INSIGHTS, 19. https://doi.org/10.1177/11786302251339881 |
| 51 | Donkor, E. S., Odoom, A., Osman, A.-H., Darkwah, S., & Kotey, F. C. N. (2024). A Systematic Review on Antimicrobial Resistance in Ghana from a One Health Perspective. Antibiotics-Basel, 13(7). https://doi.org/10.3390/antibiotics13070662 |
| 52 | Dovie, D. B. K., Dzodzomenyo, M., & Ogunseitan, O. A. (2017). Sensitivity of health sector indicators’ response to climate change in Ghana. Science of the Total Environment, 574, 837–846. https://doi.org/10.1016/j.scitotenv.2016.09.066 |
| 53 | Dsani, J. K., Johnson, S. A. M., Yasobant, S., & Bruchhausen, W. (2025). Intersectoral collaboration in zoonotic disease surveillance and response: A One Health study in the Greater Accra metropolitan area of Ghana. One Health, 21. https://doi.org/10.1016/j.onehlt.2025.101137 |
| 54 | Eger, E., Homeier-Bachmann, T., Adade, E., Dreyer, S., Heiden, S. E., Lübcke, P. K., Tawiah, P. O., Sylverken, A. A., Knauf, S., & Schaufler, K. (2024). Carbapenem- and cefiderocol-resistant Enterobacterales in surface water in Kumasi, Ashanti Region, Ghana. JAC-Antimicrobial Resistance, 6(2). https://doi.org/10.1093/jacamr/dlae021 |
| 55 | El-Duah, P., Dei, D., Binger, T., et al. (2020). Detection and genomic characterization of hepatitis E virus genotype 3 from pigs in Ghana, Africa. One Health Outlook, 2, 10. https://doi.org/10.1186/s42522-020-00018-3 |
| 56 | Emikpe, B. O., Ahmed, G. M., Akunzule, A. N., Hanson-Nortey, N. N., Awin, P., Quartey, Q., Anang, H. N. A., Agamba, A., Bayuo, R., & Okornor, R. (2024). Coordinated response to anthrax outbreaks in the Upper East Region of Ghana; the role of One Health approach. Pan African Medical Journal One Health, 13. https://doi.org/10.11604/pamj-oh.2024.13.11.42004 |
| 57 | Emikpe, B. O., Asare, D. A., Tasiame, W., Segbaya, S., Takyi, P. N., & Allegye-Cudjoe, E. (2024). Rabies control in Ghana: Stakeholders interventions, challenges and opportunities. HEALTH SCIENCE REPORTS, 7(9). https://doi.org/10.1002/hsr2.70112 |
| 58 | Emikpe, B. O., Tachie, H. F., Takyi, P. N., & Asare, D. A. (2024). One health and sustainable agricultural system in the 21st century. Pan African Medical Journal One Health, 15. https://doi.org/10.11604/pamj-oh.2024.15.11.43594 |
| 59 | Environmental Sanitation Policy (Revised 2009) |
| 60 | Eric Benbow, M.; Kimbirauskas, Ryan; McIntosh, Mollie D.; Williamson, Heather; Quaye, Charles; Boakye, Daniel et al. (2014): Aquatic macroinvertebrate assemblages of Ghana, West Africa: understanding the ecology of a neglected tropical disease. In EcoHealth 11 (2), pp. 168–183. DOI: 10.1007/s10393-013-0886-7. |
| 61 | Falgenhauer, Linda; Imirzalioglu, Can; Oppong, Kwabena; Akenten, Charity Wiafe; Hogan, Benedikt; Krumkamp, Ralf et al. (2018): Detection and Characterization of ESBL-Producing Escherichia coli From Humans and Poultry in Ghana. In Frontiers in microbiology 9, p. 3358. DOI: 10.3389/fmicb.2018.03358. |
| 62 | Fisheries & Aquaculture Act, 2025 (Act 1146) |
| 63 | Foli, S., Ros-Tonen, M. A. F., Reed, J., & Sunderland, T. C. (2018). Natural Resource Management Schemes as Entry Points for Integrated Landscape Approaches: Evidence from Ghana and Burkina Faso. Environmental Management, 62(1), 82–97. https://doi.org/10.1007/s00267-017-0866-8 |
| 64 | Food & Agriculture Sector Development Policy (FASDEP II) |
| 65 | Forestry Development Master Plan (2016–2036) |
| 66 | Ghana Agricultural Investment Plan (GhAIP) (2018–2021) |
| 67 | Ghana Forest & Wildlife Policy |
| 68 | Ghana Livestock Development Policy & Strategy |
| 69 | Ghana National Action Plan for Antimicrobial Use & Resistance (2017–2021) |
| 70 | Ghana National Aquaculture Development Plan |
| 71 | Ghana National Climate Change Master Plan – Action Programme |
| 72 | Ghana National Climate Change Policy |
| 73 | Ghana National Health Research Agenda (2015–2019) |
| 74 | Ghana Poverty Reduction Strategy (GPRS I) (2003–2005) |
| 75 | Ghana REDD+ Strategy (2016–2035) |
| 76 | Ghana Shared Growth & Development Agenda (GSGDA II) (2014–2017) |
| 77 | Ghana Shared Growth and Development Agenda (GSGDA) (2010–2013) |
| 78 | Ghana’s Intended Nationally Determined Contribution (INDC) |
| 79 | Gizamba, J. M., & Mugisha, L. (2023). Leptospirosis in humans and selected animals in Sub-Saharan Africa, 2014–2022: A systematic review and meta-analysis. BMC Infectious Diseases, 23(1). https://doi.org/10.1186/s12879-023-08574-5 |
| 80 | Goh, E. V., Sobratee-Fajurally, N., Allegretti, A., Sardeshpande, M., Mustafa, M., Azam-Ali, S. H., Omari, R., Schott, J., Chimonyo, V. G. P., Weible, D., Mutalemwa, G., Mabhaudhi, T., & Massawe, F. (2024). Transforming food environments: A global lens on challenges and opportunities for achieving healthy and sustainable diets for all. Frontiers in Sustainable Food Systems, 8. https://doi.org/10.3389/fsufs.2024.1366878 |
| 81 | Growth & Poverty Reduction Strategy (GPRS II) (2006–2009) |
| 82 | Guidelines for Handling Foodborne Disease Outbreaks |
| 83 | Hausermann, H. E., Tschakert, P., Smithwick, E. A. H., Ferring, D., Amankwah, R. K., Klutse, E. Y., Hagarty, J., & Kromel, L. (2012). Contours of risk: Spatializing human behaviors to understand disease dynamics in changing landscapes. EcoHealth, 9(3), 251–255. https://doi.org/10.1007/s10393-012-0780-8 |
| 84 | Hayman, David T. S.; Yu, Meng; Crameri, Gary; Wang, Lin-Fa; Suu-Ire, Richard; Wood, James L. N.; Cunningham, Andrew A. (2012): Ebola virus antibodies in fruit bats, Ghana, West Africa. In Emerging infectious diseases 18 (7), pp. 1207–1209. DOI: 10.3201/eid1807.111654. |
| 85 | Health Sector Medium-Term Development Plan (HSMTDP) |
| 86 | Hein, W., Aglanu, L. M., Mensah-Sekyere, M., Harant, A., Brinkel, J., Lamshöft, M. M., Lorenz, E., Eibach, D., & Amuasi, J. H. (2022). Fighting Antimicrobial Resistance: Development and Implementation of the Ghanaian National Action Plan (2017–2021). Antibiotics, 11(5). https://doi.org/10.3390/antibiotics11050613 |
| 87 | Hendrickx, E., Thomas, L.F., Dorny, P. et al. (2019). Epidemiology of Taenia saginata taeniosis/cysticercosis: A systematic review of the distribution in West and Central Africa. Parasites & Vectors, 12(1), 324. https://doi.org/10.1186/s13071-019-3584-7 |
| 88 | Hetherington, J. B., Wiethoelter, A. K., Negin, J. A., & Mor, S. M. (2017). Livestock ownership, animal source foods and child nutritional outcomes in seven rural village clusters in Sub-Saharan Africa. Agriculture and Food Security, 6(1). https://doi.org/10.1186/s40066-016-0079-z |
| 89 | Hodgson, J., Twieku, G., Quarcoo, G., Armah, E. O., Osei-Atweneboana, M. Y., & Armoo, S. (2024). Toward the elimination of NTDs: Application of cost-effective and sensitive molecular environmental surveillance tools—A pilot study. Frontiers in Parasitology, 3. https://doi.org/10.3389/fpara.2024.1340161 |
| 90 | Hunter J. M. (2003). Inherited burden of disease: agricultural dams and the persistence of bloody urine (Schistosomiasis hematobium) in the Upper East Region of Ghana, 1959-1997. Social science & medicine (1982), 56(2), 219–234. https://doi.org/10.1016/s0277-9536(02)00021-7 |
| 91 | Huntington, H., Stevens, C., Seybolt, C., Carlson, S., Tobiason, A., Daut, E., & Bouvier, I. (2024). Applications of implementation science in integrated conservation plus health programs: Improved learning to achieve environmental and health objectives. Plos Climate, 3(5). https://doi.org/10.1371/journal.pclm.0000268 |
| 92 | Integrated Disease Surveillance & Response (IDSR) 2002 |
| 93 | Investing for Food and Jobs (IFJ) (2018–2021) |
| 94 | Jagustovic, R., Zougmore, R. B., Kessler, A., Ritsema, C. J., Keesstra, S., & Reynolds, M. (2019). Contribution of systems thinking and complex adaptive system attributes to sustainable food production: Example from a climate-smart village. AGRICULTURAL SYSTEMS, 171, 65–75. https://doi.org/10.1016/j.agsy.2018.12.008 |
| 95 | Jephcott, Freya L.; Wood, James L. N.; Cunningham, Andrew A. (2017): Facility-based surveillance for emerging infectious diseases; diagnostic practices in rural West African hospital settings: observations from Ghana. In Philosophical transactions of the Royal Society of London. Series B, Biological sciences 372 (1725). DOI: 10.1098/rstb.2016.0544. |
| 96 | Jimah, T., & Ogunseitan, O. A. (2020). National Action Plan on Antimicrobial Resistance: Stakeholder analysis of implementation in Ghana. Journal of Global Health Reports, 4. https://doi.org/10.29392/001c.13695 |
| 97 | Johnson, S. A. M., Asmah, R. H., Awuni, J. A., Tasiame, W., Mensah, G. I., Pawȩska, J. T., Weyer, J., Hellferscee, O., & Thompson, P. N. (2023). Evidence of Rift Valley Fever Virus Circulation in Livestock and Herders in Southern Ghana. Viruses, 15(6). https://doi.org/10.3390/v15061346 |
| 98 | Johnson, S. A. M., Kaneene, J. B., Asare-Dompreh, K., Tasiame, W., Mensah, G. I., Afakye, K., Simpson, S. V., & Addo, K. A. (2019). Seroprevalence of Q fever in cattle, sheep and goats in the Volta region of Ghana. Veterinary Medicine and Science, 5(3), 402–411. https://doi.org/10.1002/vms3.160 |
| 99 | Kamere, N., Garwe, S. T., Akinwotu, O. O., Tuck, C., Krockow, E. M., Yadav, S., Olawale, A. G., Diyaolu, A. H., Munkombwe, D., Muringu, E., Muro, E. P., Kaminyoghe, F., Ayotunde, H. T., Omoniyei, L., Lawal, M. O., Barlatt, S. H. A., Makole, T. J., Nambatya, W., Esseku, Y., … Ashiru-Oredope, D. (2022). Scoping Review of National Antimicrobial Stewardship Activities in Eight African Countries and Adaptable Recommendations. ANTIBIOTICS-BASEL, 11(9). https://doi.org/10.3390/antibiotics11091149 |
| 100 | Kleiman, G., Anenberg, S. C., Chafe, Z. A., Appiah, D. C., Assefa Woldeamanuel, T., Bizberg, A., Coombes, T., Cuestas, D., Henze, D. K., & Kessler, A. (2022). Enhanced Integration of Health, Climate, and Air Quality Management Planning at the Urban Scale. Frontiers in Sustainable Cities, 4. https://doi.org/10.3389/frsc.2022.934672 |
| 101 | Klinkenberg, E., McCall, P. J., Wilson, M. D., Akoto, A. O. Y., Amerasinghe, F. P., Bates, I., Verhoeff, F. H., Barnish, G., & Donnelly, M. J. (2006). Urban malaria and anaemia in children: A cross-sectional survey in two cities of Ghana. Tropical Medicine and International Health, 11(5), 578–588. https://doi.org/10.1111/j.1365-3156.2006.01609.x |
| 102 | Koduah, A., Gyansa-Lutterodt, M., Hedidor, G. K., Sekyi-Brown, R., Asiedu-Danso, M., Asare, B. A., Ackon, A. A., & Annan, E. A. (2021). Antimicrobial resistance national level dialogue and action in Ghana: Setting and sustaining the agenda and outcomes. ONE HEALTH OUTLOOK, 3(1). https://doi.org/10.1186/s42522-021-00051-w |
| 103 | Kusters, K., Buck, L. E., de Graaf, M., Minang, P. A., van Oosten, C. J., & Zagt, R. J. (2018). Participatory Planning, Monitoring and Evaluation of Multi-Stakeholder Platforms in Integrated Landscape Initiatives. Environmental Management, 62(1), 170–181. https://doi.org/10.1007/s00267-017-0847-y |
| 104 | Kwarteng, P., Nyaaba, E., Honlah, E., Serbeh, R., & Abass, K. (2025). Living with familiar hazards: Flood experiences of urban households and health implications in Ghana. ENVIRONMENTAL HAZARDS-HUMAN AND POLICY DIMENSIONS. https://doi.org/10.1080/17477891.2025.2534643 |
| 105 | Kwasi Addo, Kennedy; Adjei, Vida Yirenkyiwaa; Mensah, Gloria Ivy; Jackson Sillah, Dolly (2016): Prevalence of Tuberculosis Infection in a Cohort of Cattle that Enters the Food Chain in Accra, Ghana using Bovigam. In Mycobact Dis 06 (05). DOI: 10.4172/2161-1068.1000229. |
| 106 | Laar, A. K., Aryeetey, R. N. O., Annan, R. A., Aryee, P. A., Amagloh, F. K., Akparibo, R., Laar, M. E., Amuna, P., & Zotor, F. B. (2017). Contribution of scaling up nutrition Academic Platforms to nutrition capacity strengthening in Africa: Local efforts, continental prospects and challenges. Proceedings of the Nutrition Society, 76(4), 524–534. https://doi.org/10.1017/S0029665117001124 |
| 107 | Lalonde, Anne-Marie; Hoenig, Donald; Wambui, Caroline (2017): Report of the Veterinary Legislation Identification Mission. Ghana. World Organization for Animal Health. Paris, France. |
| 108 | Land Use & Spatial Planning Act (No. 925) |
| 109 | Lawal, O. U., Ayobami, O., Abouelfetouh, A., Mourabit, N., Kaba, M., Egyir, B., Abdulgader, S. M., & Shittu, A. O. (2022). A 6-Year Update on the Diversity of Methicillin-Resistant Staphylococcus aureus Clones in Africa: A Systematic Review. FRONTIERS IN MICROBIOLOGY, 13. https://doi.org/10.3389/fmicb.2022.860436 |
| 110 | Leach, M., Bett, B., Said, M., Bukachi, S., Sang, R., Anderson, N., Machila, N., Kuleszo, J., Schaten, K., Dzingirai, V., Mangwanya, L., Ntiamoa-Baidu, Y., Lawson, E., Amponsah-Mensah, K., Moses, L. M., Wilkinson, A., Grant, D. S., & Koninga, J. (2017). Local disease—Ecosystem—Livelihood dynamics: Reflections from comparative case studies in Africa. PHILOSOPHICAL TRANSACTIONS OF THE ROYAL SOCIETY B-BIOLOGICAL SCIENCES, 372(1725). https://doi.org/10.1098/rstb.2016.0163 |
| 111 | Livestock Development in Ghana: Policies & Strategies |
| 112 | Long-term National Development Plan of Ghana (2018–2057) |
| 113 | Manual for Foodborne Disease Surveillance in Ghana |
| 114 | Mbilo, C., Coetzer, A., Bonfoh, B., Angot, A., Bebay, C., Cassamá, B., de Benedictis, P., Ebou, M. H., Gnanvi, C., & Kallo, V. (2021). Dog rabies control in West and Central Africa: A review. Acta Tropica, 224. https://doi.org/10.1016/j.actatropica.2020.105459 |
| 115 | McIntosh, Mollie; Williamson, Heather; Benbow, M. Eric; Kimbirauskas, Ryan; Quaye, Charles; Boakye, Daniel et al. (2014): Associations between Mycobacterium ulcerans and aquatic plant communities of West Africa: implications for Buruli ulcer disease. In EcoHealth 11 (2), pp. 184–196. DOI: 10.1007/s10393-013-0898-3. |
| 116 | Medium Term Agriculture Sector Investment Plan (METASIP) (2011–2015) |
| 117 | Medium-Term Agricultural Sector Investment Plan II (2014–2017) |
| 118 | Mensah, G. I., Adjei, V. Y., Vicar, E. K., Atsu, P. S., Blavo, D. L., Johnson, S. A. M., & Addo, K. K. (2022). Safety of Retailed Poultry: Analysis of Antibiotic Resistance in Escherichia coli From Raw Chicken and Poultry Fecal Matter From Selected Farms and Retail Outlets in Accra, Ghana. Microbiology insights, 15, 11786361221093278. https://doi.org/10.1177/11786361221093278. |
| 119 | Mensah, G. T., Ayeh-Kumi, P. F. K., Annang, A. K., Owusu-Frimpong, I., Niampoma, S., & Brown, C. A. (2023). Molecular epidemiology of Cryptosporidium species in Kpong and its environs, Ghana. PLOS ONE, 18(2). https://doi.org/10.1371/journal.pone.0281216 |
| 120 | Meyer, M., Melville, D. W., Baldwin, H. J., Wilhelm, K., Nkrumah, E. E., Badu, E. K., Oppong, S. K., Schwensow, N., Stow, A., Vallo, P., Corman, V. M., Tschapka, M., Drosten, C., & Sommer, S. (2024). Bat species assemblage predicts coronavirus prevalence. NATURE COMMUNICATIONS, 15(1). https://doi.org/10.1038/s41467-024-46979-1 |
| 121 | Min, K., Hwang, J., Schneider, M. C., So, Y., Lee, J. Y., & Cho, S. il. (2021). An exploration of the protective effect of rodent species richness on the geographical expansion of lassa fever in West Africa. PLOS Neglected Tropical Diseases, 15(2). https://doi.org/10.1371/journal.pntd.0009108 |
| 122 | Ministry of Food and Agriculture (MOFA) National Medium-Term Development Plan (2014–2017) |
| 123 | Ministry of Water Resources, Works and Housing (MWRWH) Sector Strategic Medium-Term Development Plan (2014–2017) |
| 124 | Mremi, I. R., George, J., Rumisha, S. F., Sindato, C., Kimera, S. I., & Mboera, L. E. G. (2021). Twenty years of integrated disease surveillance and response in Sub-Saharan Africa: challenges and opportunities for effective management of infectious disease epidemics. One health outlook, 3(1), 22. https://doi.org/10.1186/s42522-021-00052-9 |
| 125 | Mtetwa, H. N., Amoah, I. D., Kumari, S. K., Bux, F. B., & Reddy, P. (2023). Surveillance of multidrug-resistant tuberculosis in sub-Saharan Africa through wastewater-based epidemiology. Heliyon, 9(8). https://doi.org/10.1016/j.heliyon.2023.e18302 |
| 126 | Mudu, P. (2021). Ambient air pollution and health in Accra, Ghana. World Health Organization. |
| 127 | Mudu, P., Nartey, B. A., Kanhai, G., Spadaro, J. V., & Fobil, J. (2022). Solid waste management and health in Accra, Ghana. World Health Organization. |
| 128 | Narh, Charles A.; Mosi, Lydia; Quaye, Charles; Dassi, Christelle; Konan, Daniele O.; Tay, Samuel C. K. et al. (2015): Source tracking Mycobacterium ulcerans infections in the Ashanti region, Ghana. In PLoS neglected tropical diseases 9 (1), e0003437. DOI: 10.1371/journal.pntd.0003437. |
| 129 | National Action Plan to Mitigate Short-lived Climate Pollutants |
| 130 | National Action Programme to Combat Drought & Desertification |
| 131 | National Biodiversity Strategy & Action Plan |
| 132 | National Biodiversity Strategy for Ghana |
| 133 | National Centre for Coordination of Early Warning & Response Mechanism Act (Act 1070) |
| 134 | National Climate Change Adaptation Strategy |
| 135 | National Climate-Smart Agriculture & Food Security Action Plan (2016–2020) |
| 136 | National Community Water & Sanitation Strategy (NCWSS) |
| 137 | National Disaster Management Organisation Act |
| 138 | National Drinking Water Quality Management Framework |
| 139 | National Environmental Policy |
| 140 | National Environmental Sanitation Strategy & Action Plan (NESSAP) |
| 141 | National Food Safety Emergency Response Plan (FoSERP) |
| 142 | National Food Safety Policy |
| 143 | National Health Policy |
| 144 | National Health Policy |
| 145 | National Implementation Plan for the Water Convention |
| 146 | National Implementation Plan of the Stockholm Convention on POPs |
| 147 | National Integrated Water Resources Management Plan |
| 148 | National Irrigation Policy |
| 149 | National Medium-Term Development Policy Framework (2022–2025) |
| 150 | National Nutrition Policy for Ghana (2013–2017) |
| 151 | National Plan of Action to Prevent, Deter & Eliminate illegal, unreported and unregulated fishing (2021–2025) |
| 152 | National Social Protection Strategy: Investing in People |
| 153 | National Vaccine Institute Act |
| 154 | National Water Policy (2007) |
| 155 | National Water Policy (2024) |
| 156 | National Wildfire Policy |
| 157 | Nigussie, A. G., Vande Velde, F., Sarba, E. J., Kumsa, B. E., & Gabriël, S. (2025). African abattoirs: A scoping review of practices, factors influencing implementation of good practices, and recommended solutions for improvement. BMC Veterinary Research, 21(1). https://doi.org/10.1186/s12917-025-04743-7 |
| 158 | Nuvey, F. S., Fink, G., Hattendorf, J., Mensah, G. I., Addo, K. K., Bonfoh, B., & Zinsstag, J. (2023). Access to vaccination services for priority ruminant livestock diseases in Ghana: Barriers and determinants of service utilization by farmers. Preventive Veterinary Medicine, 215. https://doi.org/10.1016/j.prevetmed.2023.105919 |
| 159 | Nuvey, F. S., Haydon, D. T., Hattendorf, J., Addo, K. K., Mensah, G. I., Fink, G., Zinsstag, J., & Bonfoh, B. (2023). Relationship between animal health and livestock farmers’ wellbeing in Ghana: Beyond zoonoses. BMC Public Health, 23(1). https://doi.org/10.1186/s12889-023-16287-2 |
| 160 | Odoom, J. K., Dzotse, E. K., Nii-Trebi, N. I., Opare, D., Akyereko, E., Attiku, K., Duker, E. O., Eshun, M., Boahene, B. B., Gberbi, E., Houphouet, E. E., Diamenu, S., Adjabeng, M., Asamoah-Frimpong, J., Ameme, D., Opare, J. K. L., & Obodai, E. (2024). Outbreak Response to Circulating Vaccine-Derived Poliovirus in Three Northern Regions of Ghana, 2019. BIOMED RESEARCH INTERNATIONAL, 2024. https://doi.org/10.1155/2024/5515777 |
| 161 | Odoom, T., Johnson, S. A. M., Tasiame, W., Ziekah, M. Y., Abuh, J. K., Anderson, B., Danso, F., Abbiw, R. K., Nuokpem, F. Y., Allegye-Cudjoe, E., Lewis, C., & Faburay, B. (2024). Serological Evidence of Potential Marburg Virus Circulation in Livestock and Dogs in Ghana. PATHOGENS, 13(11). https://doi.org/10.3390/pathogens13110917 |
| 162 | Oduoye, M. O., Scott, G. Y., Dave, T., Bolanle, A. H. H., Mwinbong, A. D., & Modupeoluwa, O. O. (2024). One health approach to mitigate anthrax in Ghana. Health Science Reports, 7(1). https://doi.org/10.1002/hsr2.1807 |
| 163 | Olaitan, M. O., Orababa, O. Q., Shittu, R. B., Obunukwu, G. M., Kade, A. E., Arowolo, M. T., Oyediran, A. A., & Yusuff, R. A. (2025). Prevalence of ESBL-producing Escherichia coli in sub-Saharan Africa: A meta-analysis using a One Health approach. One Health, 20. https://doi.org/10.1016/j.onehlt.2025.101090 |
| 164 | Olu-Taiwo, M.A., Egyir, B., Owusu-Nyantakyi, C. et al. (2025) Molecular characterization of multidrug-resistant Escherichia coli in the Greater Accra Region, Ghana: a ‘One Health’ approach. One Health Outlook 7, 31 . https://doi.org/10.1186/s42522-025-00154-8 |
| 165 | Osisiogu, E. U., Singh, B., Feglo, P. K., & Duedu, K. O. (2024). Detection of PhoP-mediated colistin resistance in Gram-negative bacteria without mcr genes in human population in the Ho Municipality, Ghana. Heliyon, 10(21). https://doi.org/10.1016/j.heliyon.2024.e39633 |
| 166 | Perez Arredondo, A. M., Yasobant, S., Bruchhausen, W., Bender, K., & Falkenberg, T. (2021). Intersectoral collaboration shaping One Health in the policy agenda: A comparative analysis of Ghana and India. One Health, 13. https://doi.org/10.1016/j.onehlt.2021.100272 |
| 167 | Pileggi, Shannon M.; Jordan, Heather; Clennon, Julie A.; Whitney, Ellen; Benbow, M. Eric; Merritt, Richard et al. (2017): Landscape and environmental influences on Mycobacterium ulcerans distribution among aquatic sites in Ghana. In PloS one 12 (4), e0176375. DOI: 10.1371/journal.pone.0176375. |
| 168 | Pinto Jimenez, C. E., Pearson, M., Hennessey, M., Nkereuwem, E., Crocker, C., Egbujo, U., Hendriks, J., Smith, S. C., Whanpuch, P., & Manongi, R. N. (2023). Awareness of antibiotic resistance: A tool for measurement among human and animal health care professionals in LMICs and UMICs. Journal of Antimicrobial Chemotherapy, 78(3), 620–635. https://doi.org/10.1093/jac/dkac424 |
| 169 | Pittiglio, C., Shadomy, S., El Idrissi, A., Soumare, B., Lubroth, J., & Makonnen, Y. (2022). Seasonality and Ecological Suitability Modelling for Anthrax (Bacillus anthracis) in Western Africa. ANIMALS, 12(9). https://doi.org/10.3390/ani12091146 |
| 170 | Plants & Fertilizer (Aflatoxin Control in Maize Grains) (Technical) Regs |
| 171 | Plants & Fertilizer Act (No. 803) |
| 172 | Policy on Antimicrobial Use & Resistance for Ghana |
| 173 | Public Health Act |
| 174 | Quarcoo, G., Boamah Adomako, L. A., Abrahamyan, A., Armoo, S., Sylverken, A. A., Addo, M. G., Alaverdyan, S., Jessani, N. S., Harries, A. D., Ahmed, H., Banu, R. A., Borbor, S., Akrong, M. O., Amonoo, N. A., Bekoe, E. M. O., Osei-Atweneboana, M. Y., & Zachariah, R. (2022). What Is in the Salad? Escherichia coli and Antibiotic Resistance in Lettuce Irrigated with Various Water Sources in Ghana. International Journal of Environmental Research and Public Health, 19(19), 12722. https://doi.org/10.3390/ijerph191912722 |
| 175 | Rajaee, M., Long, R. N., Renne, E. P., & Basu, N. (2015). Mercury Exposure Assessment and Spatial Distribution in A Ghanaian Small-Scale Gold Mining Community. International journal of environmental research and public health, 12(9), 10755–10782. https://doi.org/10.3390/ijerph120910755 |
| 176 | Rajaee, M., Obiri, S. A., Green, A., Long, R. N., Cobbina, S. J., Nartey, V. K., Buck, D. G., Antwi, E., & Basu, N. (nil). (2015). Integrated Assessment of Artisanal and Small-Scale Gold Mining In Ghana—Part 2: Natural Sciences Review. International Journal of Environmental Research and Public Health, 12(8), 8971–9011. https://doi.org/10.3390/ijerph120808971 |
| 177 | Regulation Establishing the Regional Food & Agriculture Agency (RFAA) |
| 178 | Riparian Buffer Zone Policy for Managing Freshwater Bodies |
| 179 | Ros-Tonen, M. A. F., Derkyi, M. A. A., & Insaidoo, T. F. G. (2014). From co-management to landscape governance: Whither Ghana’s modified taungya system? Forests, 5(12), 2996–3021. https://doi.org/10.3390/f5122996 |
| 180 | Ohene, S. A., Klenyuie, W., & Sarpeh, M. (2016). Assessment of the response to cholera outbreaks in two districts in Ghana. |
| 181 | Sackar, S. A., Apprey, C., Aduku, L. N. E., Thow, A. M., & Annan, R. (2023). Operationalising multi-sectoral food- and nutrition-related policies to curb the rise in obesity in Ghana. Public Health Nutrition, 26(12), 3230–3238 |
| 182 | Sampane-Donkor, E. S., Odoom, A., Osman, A. H., Darkwah, S., & Kotey, F. C. N. (2025). A systematic review and meta-analysis on antibiotic resistance genes in Ghana. BMC Medical Genomics, 18(1). https://doi.org/10.1186/s12920-024-02050-y |
| 183 | Sarkodie-Addo, P., Aglomasa, B. C., & Sampane-Donkor, E. S. (2025). Prevalence and antimicrobial resistance patterns of nontyphoidal Salmonella in Ghana: A systematic review and meta-analysis. Tropical Medicine and Health, 53(1). https://doi.org/10.1186/s41182-025-00731-7 |
| 184 | Sector Medium-Term Development Plan (2014–2017) – Ministry of Fisheries and Aquaculture (MoFA) |
| 185 | Sekabira, H., Tepa-Yotto, G. T., Tamó, M., Djouaka, R. F., Dalaa, M. A., Damba, O. T., Yeboah, S., Obeng, F. A., Asare, R. K., & Abdoulaye, T. (2023). Socio-economic determinants for the deployment of Climate-Smart One-Health innovations. A meta-analysis approach prioritizing Ghana and Benin. PLOS Sustainability and Transformation, 2(3). https://doi.org/10.1371/journal.pstr.0000052 |
| 186 | Somda, N. S., Adesoji, T. O., Tetteh-Quarcoo, P. B., & Sampane-Donkor, E. S. (2025). A Systematic Review and Meta-Analysis on the Presence of Escherichia coli O157:H7 in Africa from a One Health Perspective. Microorganisms, 13(4). https://doi.org/10.3390/microorganisms13040902 |
| 187 | Sova, C. A.; Chaudhury, A. S.; Nelson, W. A.; Nutsukpo, D. K.; Zougmoré, R. (2014): Climate Change Adaptation Policy in Ghana. Priorities for the Agriculture Sector. CGIAR Research Program on Climate Change, Agriculture and Food Security. Copenhagen, Denmark (Working Paper, 68). Available online at www.ccafs.cgiar.org. |
| 188 | Specialist Health Training & Plant Medicine Research Act |
| 189 | Srivastava, D., Kutikuppala, L. V. S., Shanker, P., Sahoo, R. N., Pattnaik, G., Dash, R., Kandi, V., Ansari, A., Mishra, S., Desai, D. N., Mohapatra, R. K., Rabaan, A. A., & Kudrat-E-Zahan, Md. (2023). The neglected continuously emerging Marburg virus disease in Africa: A global public health threat. HEALTH SCIENCE REPORTS, 6(11). https://doi.org/10.1002/hsr2.1661 |
| 190 | Strategic Plan for Malaria Control in Ghana 2008-2015 |
| 191 | Suu-Ire, R. D., Obodai, E., Bonney, K. J. H., Bel-Nono, S. O., Ampofo, W. K., & Kelly, T. R. (2021). Viral Zoonoses of National Importance in Ghana: Advancements and Opportunities for Enhancing Capacities for Early Detection and Response. Journal of Tropical Medicine, 2021. https://doi.org/10.1155/2021/8938530 |
| 192 | Tasiame, W., Emikpe, B. O., Folitse, R. D., Fofie, C. O., Burimuah, V., Johnson, S. A. M., Awuni, J. A., Afari, E. A., Yebuah, N. M. N., & Wurapa, F. (2016). The prevalence of brucellosis in cattle and their handlers in North Tongu District of Volta Region, Ghana. African Journal of Infectious Diseases, 10(2), 111–117. https://doi.org/10.21010/ajid.v10i2.6 |
| 193 | The Health Sector: National Medium-Term Development Policy Framework (2014–2017) |
| 194 | Thye, T., Krumkamp, R., Lusingu, J. P. A., Ofori, L. A., Minja, D. T. R., Flieger, A., Gesase, S., Phillips, R., Simon, S., Obiri-Danso, K., Akenten, C. W., Mbwana, J., Paintsil, E., Ascofare, O. M., Jaeger, A., Lamshoeft, M., Eibach, D., Loag, W., Berg, S., … Dekker, D. (2025). Non-typhoidal Salmonella transmission reservoirs in Sub-Saharan Africa: A genomic assessment from a one health perspective. ANTIMICROBIAL RESISTANCE AND INFECTION CONTROL, 14(1). https://doi.org/10.1186/s13756-025-01561-2 |
| 195 | Tingan, T. K., Mensah, G. I., Agyekum, E. B., Amanor, I. B., Addo, S. O., Ayamdoo, Y. I., Duah, M. S., Mosi, L., & Addo, K. K. (2022). Non-tuberculous mycobacteria, not Mycobacterium bovis, are a significant cause of TB-like lesions observed in slaughtered cattle in Ghana. IJID Regions, 3, 8–14. https://doi.org/10.1016/j.ijregi.2022.02.004 |
| 196 | Traditional Medicine Practice Act |
| 197 | Triantafyllidis, C. P., Koppelaar, R. H. E. M., Wang, X., van Dam, K. H., & Shah, N. G. (2018). An integrated optimisation platform for sustainable resource and infrastructure planning. Environmental Modelling and Software, 101, 146–168. https://doi.org/10.1016/j.envsoft.2017.11.034 |
| 198 | Valeix S. F. (2018). One Health Integration: A Proposed Framework for a Study on Veterinarians and Zoonotic Disease Management in Ghana. Frontiers in veterinary science, 5, 85. https://doi.org/10.3389/fvets.2018.00085 |
| 199 | Verguet, S., Limasalle, P., Chakrabarti, A., et al. (2020). The Broader Economic Value of School Feeding Programs in Low- and Middle-Income Countries: Estimating the Multi-Sectoral Returns to Public Health, Human Capital, Social Protection, and the Local Economy. Frontiers in Public Health, 8, 587046. https://doi.org/10.3389/fpubh.2020.587046 |
| 200 | Volta Basin Water Charter |
| 201 | Waldman, Linda; Gadzekpo, Audrey; Macgregor, Hayley (2015): Responding to uncertainty. Bats and the construction of disease risk in Ghana. Brighton, UK: STEPS Centre (STEPS Working Paper, 80). |
| 202 | Wallace, Robert G.; Bergmann, Luke; Kock, Richard; Gilbert, Marius; Hogerwerf, Lenny; Wallace, Rodrick; Holmberg, Mollie (2015): The dawn of Structural One Health: A new science tracking disease emergence along circuits of capital. In Social Science & Medicine 129, pp. 68–77. DOI: 10.1016/j.socscimed.2014.09.047. |
| 203 | Water Sector Strategic Development Plan (2012–2025) |
| 204 | Wildlife Resources Management Act (Act 1115) |
| 205 | Williamson, Heather R.; Benbow, Mark E.; Nguyen, Khoa D.; Beachboard, Dia C.; Kimbirauskas, Ryan K.; McIntosh, Mollie D. et al. (2008): Distribution of Mycobacterium ulcerans in buruli ulcer endemic and non-endemic aquatic sites in Ghana. In PLoS neglected tropical diseases 2 (3), e205. DOI: 10.1371/journal.pntd.0000205. |
| 206 | Wolf, Meike (2015): Is there really such a thing as “one health”? Thinking about a more than human world from the perspective of cultural anthropology. In Social Science & Medicine 129, pp. 5–11. DOI: 10.1016/j.socscimed.2014.06.018. |
| 207 | Wood, James L. N.; Leach, Melissa; Waldman, Linda; Macgregor, Hayley; Fooks, Anthony R.; Jones, Kate E. et al. (2012): A framework for the study of zoonotic disease emergence and its drivers: spillover of bat pathogens as a case study. In Philosophical transactions of the Royal Society of London. Series B, Biological sciences 367 (1604), pp. 2881–2892. DOI: 10.1098/rstb.2012.0228. |
| 208 | World Bank. (2022, January). Pandemic Preparedness Assessment in Ghana. https://documents1.worldbank.org/curated/en/099510106132239405/pdf/P1753530e2bb160c10b1f101f1f958a0732.pdf |
| 209 | World Health Organization (2017). Joint External Evaluation of IHR Core Capacities of the Republic of Ghana. Geneva: World Health Organization; 2017. Licence: CC BY-NC-SA 3.0 IGO. |
| 210 | World Health Organization (2021). Developing public health strategies for artisanal and small-scale mining within the Minamata Convention on Mercury: findings and lessons learned from country workshops. Geneva: World Health Organization; 2021. Licence: CC BY-NC-SA 3.0 IGO. |
| 211 | World Health Organization (2025). Joint external evaluation of the International Health Regulations (2005) core capacities of Ghana: mission report, 10-14 February 2025. Geneva: World Health Organization; 2025. Licence: CC BY-NC-SA 3.0 IGO |
| 212 | World Health Organization (Ed.) (2016): Report on One Health Technichal and Ministerial Meeting to Address Zoonotic Diseases and Related Public Health Threats. Radisson Blu Hotel, Dakar (Senegal), 8-11 November. World Health Organization; Food and Agriculture Organization of the United Nations; World Animal Health Organization; West African Health Organization; Regional Centre for Animal Health of the Economic Community of West African States; United States Agency for International Development; The World Bank. Available online at https://www.afro.who.int/publications/report-one-health-technical-and-ministerial-meeting-address-zoonotic-diseases-and. |
| 213 | World Health Organization. (2020). Achieving quality health services for all through better water sanitation and hygiene: lessons from three African countries. World Health Organization. |
| 214 | World Health Organization. (2022). WHO Urban Health Initiative in Accra, Ghana: summary of project results. World Health Organization. |
| 215 | Wurapa, F., Afari, E., Ohuabunwo, C., Sackey, S., Clerk, C., Kwadje, S., Yebuah, N., Amankwa, J., Amofah, G., & Appiah-Denkyira, E. (2011). One Health concept for strengthening public health surveillance and response through Field Epidemiology and Laboratory Training in Ghana. The Pan African medical journal, 10 Supp 1(Suppl 1), 6. |
| 216 | Yeboah, J. A., Offih-Kyei, W., Danso-Coffie, C. K., Boafo, E. K., Banahene, P., Yeboah, R., Futagbi, G., Bimi, L., & Oduro, D. (2024). Assessment of enteric helminth parasites in bushmeat in Ghana. International Journal for Parasitology: Parasites and Wildlife, 25. https://doi.org/10.1016/j.ijppaw.2024.101005 |
| 217 | Yeboah, R., Sylverken, A. A., Owusu, M., et al. (2021) (2021). Sero-molecular epidemiology of hepatitis E virus in pigs and human contacts in Ghana. One Health Outlook, 3(1), 13. https://doi.org/10.1186/s42522-021-00043-w |
| 218 | Yevutsey, Saviour Kwame; Buabeng, Kwame Ohene; Aikins, Moses; Anto, Berko Panyin; Biritwum, Richard B.; Frimodt-Møller, Niels; Gyansa-Lutterodt, Martha (2017): Situational analysis of antibiotic use and resistance in Ghana: policy and regulation. In BMC public health 17 (1), p. 896. DOI: 10.1186/s12889-017-4910-7. |
| 219 | Zhou, H., Baltenweck, I., Dekkers, J. C. M., Gallardo, R. A., Kayang, B. B., Kelly, T. R., Msoffe, P. L. M., Muhairwa, A. P., Mushi, J. R., & Naazie, A. (2024). Feed the Future Innovation Lab for Genomics to Improve Poultry: A holistic approach to improve indigenous chicken production focusing on resilience to Newcastle disease. World’s Poultry Science Journal, 80(2), 273–297. https://doi.org/10.1080/00439339.2024.2321350 |
| 220 | Zornu, J., Oyih, M., Binde, M., Viglo, J., Agbekpornu, H., Nkansa, M., Tavornpanich, S., Norheim, K., Brun, E., & Cudjoe, K. S. (2023). Stakeholder perspectives on the 2023 Ghana National Aquaculture Development Plan: An integration within the ecosystem approach framework. Aquaculture, Fish And Fisheries, 3(6), 459–471. https://doi.org/10.1002/aff2.135 |

| Table 3. Thematic clustering: OH Narratives, using parameters for each narrative | |
| --- | --- |
| **Narrative** | **Record ID, as in Table 1 (Appendix B)** |
| **1 “A holistic, integrated approach to deal with complex interactions”** | |
| (1) Interdependence of health systems | 2,7,11,12,22,24,34,40,42,43,48,49,50,53,56,57,59,61,62,64,65,66,67,68,69,70,71,72,73,74,75,76,78,81,82,85,86,92,93,95,96,99,102,107,108,112,113,117,122,123,124,126,127,129,131,132,134,135,136,137,138,139,140,141,142,143,144,145,146,147,148,149,150,151,152,153,154,155,156,160,162,166,172,177,178,180,181,184,187,190,193,196,199,200,204,208,209,210,211,213,214,218,220 |
| (2) Collaboration between health practitioners | 1,6,8,17,25,27,55,97,98,114,158,168,192,198,215 |
| (3) Integration of disciplines | 3,4,10,13,14,15,18,19,20,21,23,26,28,29,30,31,32,33,35,36,37,38,39,41,44,45,46,47,51,52,54,58,60,63,79,80,83,84,87,88,89,90,91,94,100,101,103,104,106,109,110,115,118,119,120,121,125,128,157,159,161,163,164,165,167,169,174,175,176,179,182,183,185,186,189,191,194,195,197,201,205,206,207,212,216,217,219 |
| **2 “A way to prevent risk and respond to crises”** | |
| (1) Surveillance and Prevention | 1,2,5,11,24,31,34,50,53,58,65,67,73,82,85,89,92,93,95,107,113,124,125,133,141,142,143,149,153,166,193,208,209,211,212,215 |
| (2) Sanitation | 4,19,21,29,36,37,49,59,127,138,140,145,147,148,155,178,200 |
| (3) Disease Emergence (including zoonotic/vector-borne diseases, infectious diseases, and antimicrobial resistance) | 6,7,8,10,12,13,14,17,18,20,25,26,27,28,30,35,38,41,44,48,51,54,55,56,57,60,61,68,69,79,83,84,86,87,88,90,91,96,97,98,99,101,102,105,109,110,111,114,115,118,119,120,121,128,144,157,158,159,160,161,162,163,164,165,167,168,169,172,174,180,182,183,185,186,189,190,191,192,194,195,198,201,205,206,207,216,217,218,219,220 |
| **3 “Economic benefits of implementing One Health approaches”** | |
| (1) Health and other socio-economic changes | 5,37,46,90,101,159,199 |
| (2) Science, Policy and Political Action | 1,2,3,4,6,7,8,11,12,19,21,22,23,24,26,27,29,30,31,33,36,38,39,40,42,43,45,47,48,49,50,52,53,56,57,58,59,63,64,65,66,67,68,69,70,71,72,73,74,75,76,78,79,80,81,85,86,87,88,89,91,92,93,94,96,99,100,102,103,104,106,107,108,109,110,111,112,114,117,118,121,122,123,124,125,126,127,129,131,132,133,134,135,136,137,138,139,140,141,142,143,144,145,146,147,148,149,150,151,152,153,154,155,156,157,158,160,161,162,163,164,166,168,169,172,174,176,177,178,179,180,181,182,183,184,185,186,187,189,190,191,193,197,198,200,201,204,208,211,212,213,214,215,218,219,220 |
| **4 “Local ecological and disease contexts”** | |
| (1) Community participation and development of community | 2,5,8,21,24,29,30,31,32,33,40,41,42,43,45,46,49,50,53,56,57,58,59,62,63,64,65,66,67,68,69,70,71,72,73,74,75,76,78,80,81,85,86,89,91,92,93,94,96,98,99,100,102,103,107,108,111,112,113,114,117,151,152,154,155,156,160,162,166,172,179,180,181,184,185,187,189,190,191,192,193,196,197,198,199,200,201,204,206,208,209,210,211,212,213,214,215 |
| (2) Behavioural factors | 1,6,10,11,13,17,18,22,23,25,26,28,36,48,55,60,83,87,106,115,157,158,168,174,195,205,216,217,218 |
| (3) Urbanization, human mobility, and population growth | 4,16,19,37,90,101,104 |
| (4) Susceptibility to disease | 20,35,38,51,61,79,109,118,119,163,165,182,186 |
| (5) Environmental Drivers | 3,7,12,14,15,39,44,47,52,54,84,88,110,120,159,161,164,167,169,175,176,178,183,194,207,219,220 |
